# Incidence, Factors, and Patient-Level Data for Spontaneous HBsAg Seroclearance: A Cohort Study of 11,264 Patients

**DOI:** 10.14309/ctg.0000000000000196

**Published:** 2020-09-15

**Authors:** Yee Hui Yeo, Tai-Chung Tseng, Tetsuya Hosaka, Chris Cunningham, James Yan Yue Fung, Hsiu J. Ho, Min-Sun Kwak, Huy N. Trinh, Teerapat Ungtrakul, Ming-Lung Yu, Mariko Kobayashi, An K. Le, Linda Henry, Jiayi Li, Jian Zhang, Tassanee Sriprayoon, Donghak Jeong, Tawesak Tanwandee, Ed Gane, Ramsey C. Cheung, Chun-Ying Wu, Anna S. Lok, Hyo-Suk Lee, Fumitaka Suzuki, Man-Fung Yuen, Jia-Horng Kao, Hwai-I Yang, Mindie H. Nguyen

**Affiliations:** 1 Department of Medicine, Stanford University Medical Center, Palo Alto, California, USA;; 2 Division of Gastroenterology and Hepatology, Department of Internal Medicine, National Taiwan University Hospital, Taipei, Taiwan;; 3 Department of Hepatology, Toranomon Hospital, Tokyo, Japan;; 4 Research Centre for Maori Health and Development, Massey University, Wellington, New Zealand;; 5 The Hepatitis Foundation of New Zealand, Whakatane, New Zealand;; 6 Department of Medicine, The University of Hong Kong, Hong Kong, China;; 7 Division of Translational Research, Taipei Veterans General Hospital, Taipei City, Taiwan;; 8 Department of Internal Medicine and Liver Research Institute, Seoul National University Hospital, Seoul, Korea;; 9 San Jose Gastroenterology, San Jose, California, USA;; 10 Faculty of Medicine and Public Health, HRH Princess Chulabhorn College of Medical Science, Chulabhorn Royal Academy, Bangkok, Thailand;; 11 Hepatobiliary Section, Department of Internal Medicine, Kaohsiung Medical University Hospital, Kaohsiung, Taiwan;; 12 Center for Liver Research, School of Medicine, Kaohsiung Medical University, Kaohsiung, Taiwan;; 13 Center for Cancer Research, Kaohsiung Medical University, Kaohsiung, Taiwan;; 14 Research Institute for Hepatology, Toranomon Hospital, Tokyo, Japan;; 15 Palo Alto Medical Foundation, Mountain View Division, Palo Alto, California, USA;; 16 Chinese Hospital, San Francisco, California, USA;; 17 School of Nursing, University of California, San Francisco, San Francisco, California, USA;; 18 Department of Medicine, Faculty of Medicine, Siriraj Hospital, Mahidol University, Bangkok, Thailand;; 19 New Zealand Liver Transplant Unit, Auckland City Hospital, Auckland, New Zealand;; 20 Department of Medicine, University of Auckland, Auckland, New Zealand;; 21 Division of Gastroenterology and Hepatology, Veterans Affairs Palo Alto Health Care System, Palo Alto, California, USA;; 22 College of Public Health, China Medical University, Taichung, Taiwan;; 23 Division of Gastroenterology and Hepatology, University of Michigan, Ann Arbor, Michigan, USA;; 24 Graduate Institute of Clinical Medicine, College of Medicine, National Taiwan University, Taipei, Taiwan;; 25 Genomics Research Center, Academia Sinica, Taipei, Taiwan;; 26 Institute of Clinical Medicine, National Yang-Ming University, Taipei, Taiwan.

## Abstract

Spontaneous hepatitis B surface antigen (HBsAg) seroclearance, the functional cure of hepatitis B infection, occurs rarely. Prior original studies are limited by insufficient sample size and/or follow-up, and recent meta-analyses are limited by inclusion of only study-level data and lack of adjustment for confounders to investigate HBsAg seroclearance rates in most relevant subgroups. Using a cohort with detailed individual patient data, we estimated spontaneous HBsAg seroclearance rates through patient and virologic characteristics.

**METHODS:**

We analyzed 11,264 untreated patients with chronic hepatitis B with serial HBsAg data from 4 North American and 8 Asian Pacific centers, with 1,393 patients with HBsAg seroclearance (≥2 undetectable HBsAg ≥6 months apart) during 106,192 person-years. The annual seroclearance rate with detailed categorization by infection phase, further stratified by hepatitis B e antigen (HBeAg) status, sex, age, and quantitative HBsAg (qHBsAg), was performed.

**RESULTS:**

The annual seroclearance rate was 1.31% (95% confidence interval: 1.25–1.38) and over 7% in immune inactive patients aged ≥55 years and with qHBsAg <100 IU/mL. The 5-, 10-, 15-, and 20-year cumulative rates were 4.74%, 10.72%, 18.80%, and 24.79%, respectively. On multivariable analysis, male (adjusted hazard ratio [aHR] = 1.66), older age (41–55 years: aHR = 1.16; >55 years: aHR = 1.21), negative HBeAg (aHR = 6.34), and genotype C (aHR = 1.82) predicted higher seroclearance rates, as did lower hepatitis B virus DNA and lower qHBsAg (*P* < 0.05 for all), and inactive carrier state.

**DISCUSSION:**

The spontaneous annual HBsAg seroclearance rate was 1.31%, but varied from close to zero to about 5% among most chronic hepatitis B subgroups, with older, male, HBeAg-negative, and genotype C patients with lower alanine aminotransferase and hepatitis B virus DNA, and qHBsAg independently associated with higher rates (see Visual Abstract, Supplementary Digital Content 2, http://links.lww.com/CTG/A367).

## INTRODUCTION

Chronic hepatitis B (CHB) affects about 257 million people worldwide (1) and can lead to cirrhosis and hepatocellular carcinoma (HCC) (2,3). Persons who have achieved hepatitis B surface antigen (HBsAg) seroclearance, the functional cure, had a significantly lower risk of HCC than those without, even in the setting of established cirrhosis (4–6). However, only a small proportion of patients will eventually experience HBsAg seroclearance spontaneously or with currently available antiviral therapies (7–12). Thus, major efforts are underway to identify new strategies and drugs that can yield higher seroclearance rates.

As the rate of spontaneous HBsAg seroclearance can vary by host and viral factors (13), it is important to inform stakeholders of detailed subgroup data for the plan and design of therapeutic trials of drugs that aim to significantly increase the rate of HBsAg seroclearance compared with the expected spontaneous rate. Because of the low rate, previous studies are limited by inadequate sample sizes and follow-up periods. In 2 recent meta-analyses, we and others have provided pooled estimates for the HBsAg seroclearance rates (12,13). However, the results, especially for subgroup analyses, were limited by a number of factors. First, there was severe heterogeneity among the studies, and adequate adjustment for confounders was not possible without individual patient-level data. Second, because of a lack of primary data, subgroup analyses were not possible for several clinically relevant variables, such as age and infection phase. Third, without individual patient data, subgroup analyses were possible for only 1 patient or 1 viral factor at a time, such as male vs female or high vs low hepatitis B virus (HBV) DNA. Therefore, to fill in current knowledge gaps, we used data from a large collaborative cohort of never-treated patients not only to determine the annual and cumulative incidence rates of spontaneous HBsAg seroclearance but also to investigate independent host and viral factors associated with spontaneous HBsAg seroclearance by detailed subgroup analyses and multivariable regressions.

## METHODS

### Data source

The current study cohort comprised patients from 4 North American and 8 Asian Pacific study centers. Data were collected using a standardized data collection form with unified variable definition at each center. Deidentified data were transmitted to the data monitoring and analysis center at Stanford University. The study was approved by the Institutional Review Board at Stanford University and at each center.

### Inclusion and exclusion criteria

The study included patients with CHB (18 years or older at baseline, not receiving HBV treatment at baseline and throughout study follow-up, and HBsAg and/or HBV DNA positive for at least 6 months), who were enrolled at each study center with serial laboratory data (including HBsAg, quantitative HBsAg [qHBsAg], hepatitis B e antigen [HBeAg], liver chemistries, and HBV DNA).

We excluded patients with any of the following: (i) hepatitis C virus, hepatitis D virus, or HIV coinfection, (ii) prior HCC, (iii) medical history of solid organ or bone marrow transplantation, immunosuppression including chemotherapy and biologics, (iv) other known liver diseases (autoimmune hepatitis, hemochromatosis, Wilson disease, and alpha-1 antitrypsin deficiency), and (v) known malignancy within 5 years of study entry date.

### Study variable definitions

Infection phase was defined as per the American Association for the Study of Liver Diseases Guidance (see Table 1, Supplementary Digital Content 1, http://links.lww.com/CTG/A366) (14). The primary outcome of this study was confirmed spontaneous HBsAg seroclearance, defined as having ≥2 negative HBsAg results ≥6 months apart in the absence of any antiviral treatment. The secondary outcome was to examine the determinants of spontaneous HBsAg seroclearance. Follow-up was from the first date patients were evaluated at the study centers for CHB. Patients were censored once they started treatment, with HBsAg seroclearance, death, or the end of follow-up as the study end point, whichever came first.

### Statistical analysis

We estimated the follow-up period in person-years by multiplying the duration of follow-up by the number of patients. We calculated the annual HBsAg seroclearance rate by dividing the number of confirmed HBsAg seroclearance events by the total follow-up period. Kaplan-Meier analysis was used to estimate the 5-, 10-, 15-, and 20-year cumulative HBsAg seroclearance rates. Log-rank tests were used to determine differences in cumulative incidence rates. To avert bias caused by the small sample size, we omitted the data from subgroups with <2 events or <20 patients. In addition, we performed separate subgroup analyses for genotype B and C patients with further stratification by age, sex, and qHBsAg.

Given that the observations were derived in clusters from each study site, we used Cox models with frailty to evaluate the HBsAg seroclearance rate (15) to allow us to model the heterogeneity across the cohorts and to account for the potential random effects at study level. Thus, a mixed-effects model was adopted, with random effects set for cohorts and fixed effects for individual patients' characteristics. We performed multivariable Cox regression to adjust for clinically relevant covariates. Variables with >10% missing data were not included in the main model. The assumption of the Cox regression was tested. For variables with violation, we added a time interaction of that variable into the model. We also assessed goodness of fit of the model by estimating the Harrell C statistics and determined the E values of variables to assess the magnitude that an unmeasured confounder would have to explain the association between the corresponding variable and spontaneous HBsAg seroclearance (16). Two-sided *P* values were calculated, with the threshold of significance set at 0.05. All statistical analyses were performed using R software (3.5.1).

## RESULTS

The study analysis included 11,264 eligible patients. The study cohort was predominately male (60.1%), Asian/Pacific Islanders/Polynesians (98.1%), noncirrhotic (97.0%), aged 55 years or younger (82.8%), and HBeAg negative (82.0%) at baseline (Table 1). Patients seen at health care centers accounted for approximately two-thirds of the study population. Patients who achieved HBsAg seroclearance during follow-up period were compared with those remained positive for HBsAg.

**Table 1.**
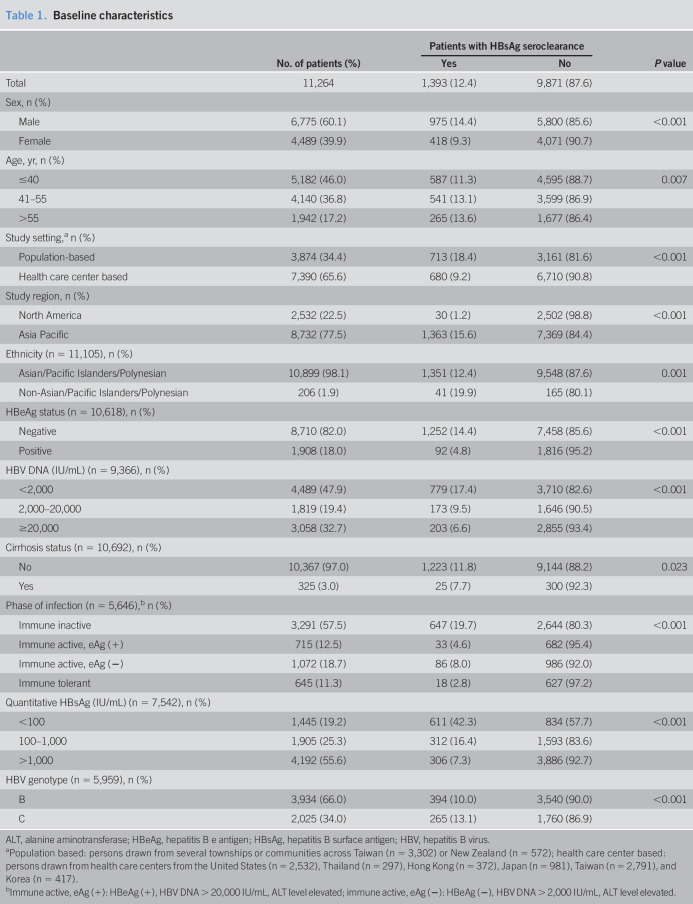
Baseline characteristics

	No. of patients (%)	Patients with HBsAg seroclearance		*P* value
		Yes	No	
Total	11,264	1,393 (12.4)	9,871 (87.6)	
Sex, n (%)				
Male	6,775 (60.1)	975 (14.4)	5,800 (85.6)	<0.001
Female	4,489 (39.9)	418 (9.3)	4,071 (90.7)	
Age, yr, n (%)				
≤40	5,182 (46.0)	587 (11.3)	4,595 (88.7)	0.007
41–55	4,140 (36.8)	541 (13.1)	3,599 (86.9)	
>55	1,942 (17.2)	265 (13.6)	1,677 (86.4)	
Study setting,^a^ n (%)				
Population-based	3,874 (34.4)	713 (18.4)	3,161 (81.6)	<0.001
Health care center based	7,390 (65.6)	680 (9.2)	6,710 (90.8)	
Study region, n (%)				
North America	2,532 (22.5)	30 (1.2)	2,502 (98.8)	<0.001
Asia Pacific	8,732 (77.5)	1,363 (15.6)	7,369 (84.4)	
Ethnicity (n = 11,105), n (%)				
Asian/Pacific Islanders/Polynesian	10,899 (98.1)	1,351 (12.4)	9,548 (87.6)	0.001
Non-Asian/Pacific Islanders/Polynesian	206 (1.9)	41 (19.9)	165 (80.1)	
HBeAg status (n = 10,618), n (%)				
Negative	8,710 (82.0)	1,252 (14.4)	7,458 (85.6)	<0.001
Positive	1,908 (18.0)	92 (4.8)	1,816 (95.2)	
HBV DNA (IU/mL) (n = 9,366), n (%)				
<2,000	4,489 (47.9)	779 (17.4)	3,710 (82.6)	<0.001
2,000–20,000	1,819 (19.4)	173 (9.5)	1,646 (90.5)	
≥20,000	3,058 (32.7)	203 (6.6)	2,855 (93.4)	
Cirrhosis status (n = 10,692), n (%)				
No	10,367 (97.0)	1,223 (11.8)	9,144 (88.2)	0.023
Yes	325 (3.0)	25 (7.7)	300 (92.3)	
Phase of infection (n = 5,646),^b^ n (%)				
Immune inactive	3,291 (57.5)	647 (19.7)	2,644 (80.3)	<0.001
Immune active, eAg (+)	715 (12.5)	33 (4.6)	682 (95.4)	
Immune active, eAg (−)	1,072 (18.7)	86 (8.0)	986 (92.0)	
Immune tolerant	645 (11.3)	18 (2.8)	627 (97.2)	
Quantitative HBsAg (IU/mL) (n = 7,542), n (%)				
<100	1,445 (19.2)	611 (42.3)	834 (57.7)	<0.001
100–1,000	1,905 (25.3)	312 (16.4)	1,593 (83.6)	
>1,000	4,192 (55.6)	306 (7.3)	3,886 (92.7)	
HBV genotype (n = 5,959), n (%)				
B	3,934 (66.0)	394 (10.0)	3,540 (90.0)	<0.001
C	2,025 (34.0)	265 (13.1)	1,760 (86.9)	

ALT, alanine aminotransferase; HBeAg, hepatitis B e antigen; HBsAg, hepatitis B surface antigen; HBV, hepatitis B virus.

a Population based: persons drawn from several townships or communities across Taiwan (n = 3,302) or New Zealand (n = 572); health care center based: persons drawn from health care centers from the United States (n = 2,532), Thailand (n = 297), Hong Kong (n = 372), Japan (n = 981), Taiwan (n = 2,791), and Korea (n = 417).

b Immune active, eAg (+): HBeAg (+), HBV DNA > 20,000 IU/mL, ALT level elevated; immune active, eAg (−): HBeAg (−), HBV DNA > 2,000 IU/mL, ALT level elevated.

### Annual spontaneous HBsAg seroclearance rates

**Figure 1.**
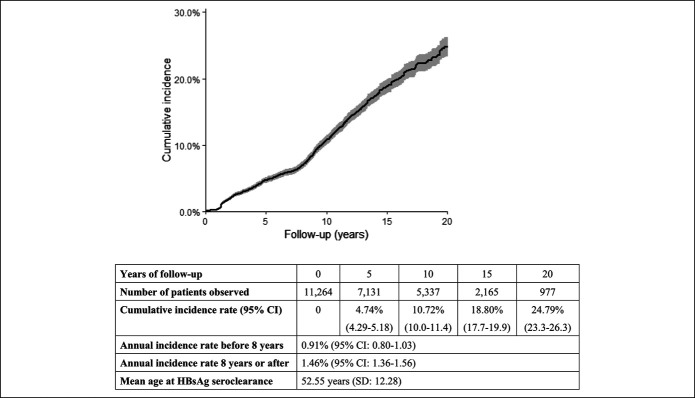
Cumulative incidence rate of spontaneous hepatitis B surface antigen (HBsAg) seroclearance. Gray shade denotes 95% confidence interval (CI) of the cumulative incidence rate.

After 106,192 person-years of follow-up, there were 1,393 patients with spontaneous HBsAg seroclearance (see Table 2, Supplementary Digital Content 1, http://links.lww.com/CTG/A366). The annual rate of spontaneous HBsAg seroclearance was 1.31% (95% confidence interval [CI]: 1.25–1.38). The mean age (±SD) at HBsAg seroclearance was 52.55 (±12.28) years (Figure 1, bottom panel).

Table 2 (see Supplementary Digital Content 1, http://links.lww.com/CTG/A366) shows the annual incidence rate of spontaneous HBsAg seroclearance stratified by sex, age, study setting, ethnicity, HBeAg status, cirrhosis, HBV DNA, qHBsAg, and HBV genotype. The annual incidence rate was higher in men, patients aged >55 years, patients seen in health care centers, those with HBV genotype C, HBV DNA ≤2,000 IU/mL, qHBsAg ≤500 IU/mL, or HBeAg negative at baseline. Table 2 demonstrates the annual incidence rate by infection phase, with further stratification by HBeAg status, sex, and age. Table 3 (see Supplementary Digital Content 1, http://links.lww.com/CTG/A366) showed the similar results with different age cutoff (≤40, 41–50, and >50 years)

**Table 2.**
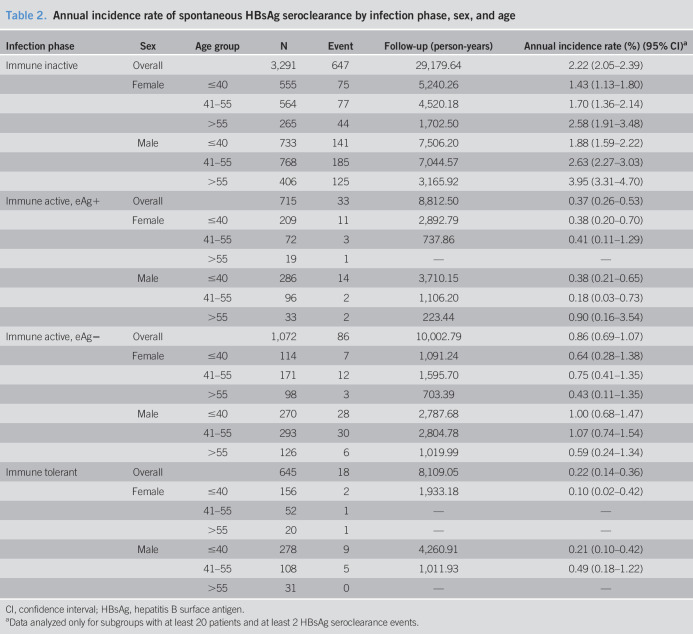
Annual incidence rate of spontaneous HBsAg seroclearance by infection phase, sex, and age

Infection phase	Sex	Age group	N	Event	Follow-up (person-years)	Annual incidence rate (%) (95% CI)^a^
Immune inactive	Overall		3,291	647	29,179.64	2.22 (2.05–2.39)
	Female	≤40	555	75	5,240.26	1.43 (1.13–1.80)
		41–55	564	77	4,520.18	1.70 (1.36–2.14)
		>55	265	44	1,702.50	2.58 (1.91–3.48)
	Male	≤40	733	141	7,506.20	1.88 (1.59–2.22)
		41–55	768	185	7,044.57	2.63 (2.27–3.03)
		>55	406	125	3,165.92	3.95 (3.31–4.70)
Immune active, eAg+	Overall		715	33	8,812.50	0.37 (0.26–0.53)
	Female	≤40	209	11	2,892.79	0.38 (0.20–0.70)
		41–55	72	3	737.86	0.41 (0.11–1.29)
		>55	19	1	—	—
	Male	≤40	286	14	3,710.15	0.38 (0.21–0.65)
		41–55	96	2	1,106.20	0.18 (0.03–0.73)
		>55	33	2	223.44	0.90 (0.16–3.54)
Immune active, eAg−	Overall		1,072	86	10,002.79	0.86 (0.69–1.07)
	Female	≤40	114	7	1,091.24	0.64 (0.28–1.38)
		41–55	171	12	1,595.70	0.75 (0.41–1.35)
		>55	98	3	703.39	0.43 (0.11–1.35)
	Male	≤40	270	28	2,787.68	1.00 (0.68–1.47)
		41–55	293	30	2,804.78	1.07 (0.74–1.54)
		>55	126	6	1,019.99	0.59 (0.24–1.34)
Immune tolerant	Overall		645	18	8,109.05	0.22 (0.14–0.36)
	Female	≤40	156	2	1,933.18	0.10 (0.02–0.42)
		41–55	52	1	—	—
		>55	20	1	—	—
	Male	≤40	278	9	4,260.91	0.21 (0.10–0.42)
		41–55	108	5	1,011.93	0.49 (0.18–1.22)
		>55	31	0	—	—

CI, confidence interval; HBsAg, hepatitis B surface antigen.

a Data analyzed only for subgroups with at least 20 patients and at least 2 HBsAg seroclearance events.

Overall, the annual incidence rates were 2.22% (95% CI: 2.05%–2.39%), 0.37% (95% CI: 0.26%–0.53%), 0.86% (95% CI: 0.69%–1.07%), and 0.22% (95% CI: 0.14%–0.36%) in immune inactive, immune active (eAg−), immune active (eAg+), and immune tolerant, respectively. Males aged 55 years or older with immune inactive infection showed the highest annual incidence rate of spontaneous HBsAg seroclearance (3.95% [95% CI: 3.31–4.70]), followed by males aged 41–55 years with immune inactive infection (2.63% [95% CI: 2.27–3.03]) and females aged 55 years or older with immune inactive infection (2.58% [95% CI: 1.91–3.48]). Subgroup analysis by infection phase, sex, age, and qHBsAg showed that both males and females aged 55 years or older with immune inactive phase and qHBsAg <100 IU/mL presented an annual incidence rate higher than 7% (see Table 4, Supplementary Digital Content 1, http://links.lww.com/CTG/A366).

### Cumulative spontaneous HBsAg seroclearance rates

Overall, the 5-, 10-, and 20-year cumulative incidence rates were 4.74% (95% CI: 4.29–5.18), 10.72% (95% CI: 10.0–11.4), and 24.79% (95% CI: 23.3–26.3), respectively (Figure 1).

**Figure 2.**
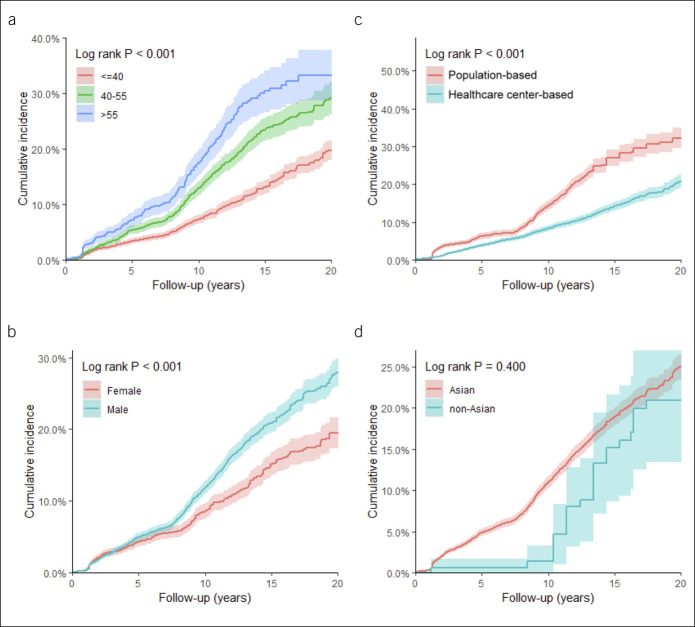
Cumulative incidence rate of spontaneous hepatitis B surface antigen seroclearance. (**a**) Age (years). Levels of significance: *P* < 0.001 (log-rank test). (**b**) Sex. Levels of significance: *P* < 0.001 (log-rank test). (**c**) Study setting. Levels of significance: *P* < 0.001 (log-rank test). (**d**) Ethnicity. Levels of significance: *P* = 0.400 (log-rank test). Color shades denote 95% confidence interval of the cumulative incidence rate. Corresponding risk tables can be found in Table 4A–D (see Supplementary Digital Content 1, http://links.lww.com/CTG/A366).

Figure 2a–d and see Table 5A–D (Supplementary Digital Content 1, http://links.lww.com/CTG/A366) show that cumulative incidence rates of spontaneous HBsAg seroclearance were significantly higher in males, older patients, and patients from population-based study settings. The 20-year cumulative incidence rates of those who were aged 40 years or younger, 41–55 years, and older than 55 years were 19.69% (95% CI: 17.89–21.49), 29.13% (95% CI: 26.18–32.09), and 33.19% (95% CI: 28.64–37.75), respectively (*P* < 0.001) (Figure 2a and see Table 5A, Supplementary Digital Content 1, http://links.lww.com/CTG/A366). The 20-year cumulative incidence rates were 27.90% (95% CI: 25.93–29.86) in males and 19.48% (95% CI: 17.32–21.64) in females (*P* < 0.001) (Figure 2b and see Table 5B, Supplementary Digital Content 1, http://links.lww.com/CTG/A366).

Data from population-based community patients had a 20-year cumulative incidence rate of 32.26% (95% CI: 29.59–34.94), which was higher than the 20.70% (95% CI: 18.89–22.52) incidence in health care center–based patients (*P* < 0.001) (Figure 2c and see Table 5C, Supplementary Digital Content 1, http://links.lww.com/CTG/A366). Further stratification by age is demonstrated in Figure 1 (see Supplementary Digital Content 1, http://links.lww.com/CTG/A366). In addition, there was no significant difference in the cumulative incidence rates between Asian/Pacific Islanders/Polynesians and non-Asian/Pacific Islanders/Polynesians patients (*P* = 0.40), but the sample size of non-Asian/Pacific Islanders/Polynesians patients was small (Figure 2D and see Table 5D, Supplementary Digital Content 1, http://links.lww.com/CTG/A366). Subgroup analyses by age and sex are shown in Figure 2 (see Supplementary Digital Content 1, http://links.lww.com/CTG/A366). Notably, the significant difference in HBsAg seroclearance rates across age groups was observed in both sexes.

### Subgroup analysis based on viral factors

**Figure 3.**
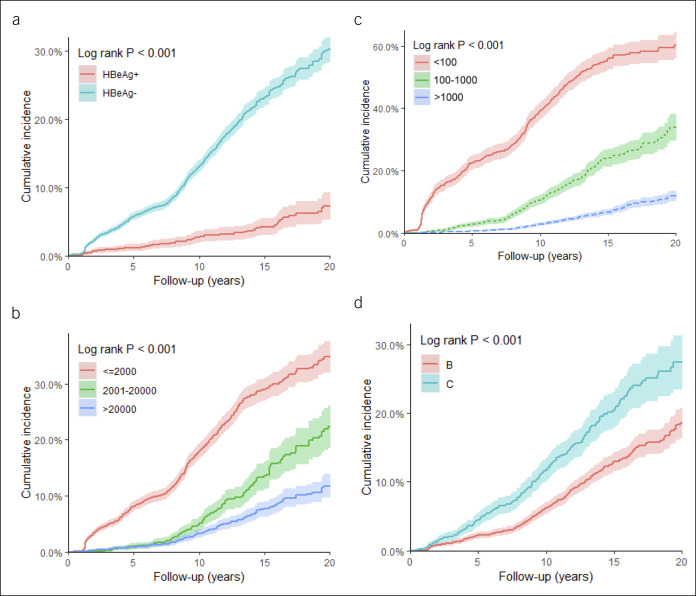
Cumulative incidence rate of spontaneous hepatitis B surface antigen (HBsAg) seroclearance. (**a**) Hepatitis B e antigen status. Levels of significance: *P* < 0.001 (log-rank test). (**b**) Hepatitis B virus DNA (IU/mL). Levels of significance: *P* < 0.001 (log-rank test). (**c**) Quantitative HBsAg (IU/mL). Levels of significance: *P* < 0.001 (log-rank test). (**d**) Genotype. Levels of significance: *P* < 0.001 (log-rank test). Color shades denote 95% confidence interval of the cumulative incidence rate. Corresponding risk tables can be found in Table 5A–D (see Supplementary Digital Content 1, http://links.lww.com/CTG/A366).

The cumulative incidence rate of HBsAg seroclearance at 20 years in patients who were HBeAg negative at baseline was 30.25% (95% CI: 28.36–32.15) compared with 7.31% (95% CI: 5.33–9.28) in those who were HBeAg positive (*P* < 0.001) (Figure 3a and see Table 6A, Supplementary Digital Content 1, http://links.lww.com/CTG/A366). When stratified by HBeAg status, the difference between age groups, HBV DNA, and qHBsAg remained significant in both HBeAg groups (see Figure 3A, C, and D, Supplementary Digital Content 1, http://links.lww.com/CTG/A366). In contrast, the difference by HBV genotype and sex was only significant in the HBeAg-negative group, and this was likely due to the smaller number of HBeAg-positive patients and the low rate of HBsAg seroclearance in this group (see Figure 3B, Supplementary Digital Content 1, http://links.lww.com/CTG/A366).

Patients with lower baseline HBV DNA levels had significantly greater 20-year cumulative incidence, with 34.76% (95% CI: 31.99–37.52), 22.29% (95% CI: 18.49–26.08), and 11.82% (95% CI: 9.80–13.83) for patients with baseline HBV DNA ≤2,000 IU/mL, 2,001–20,000 IU/mL, and >20,000 IU/mL, respectively (*P* < 0.001) (Figure 3b and see Table 6B, Supplementary Digital Content 1, http://links.lww.com/CTG/A366).

The 20-year cumulative incidence rates for patients with qHBsAg <100 IU/mL, 100–1,000 IU/mL, and >1,000 IU/mL were 60.25% (95% CI: 56.05–64.46), 33.82% (95% CI: 29.53–38.11), and 12.00% (95% CI: 10.33–13.67), respectively (*P* < 0.001) (Figure 3c and see Table 6C, Supplementary Digital Content 1, http://links.lww.com/CTG/A366).

**Figure 4.**
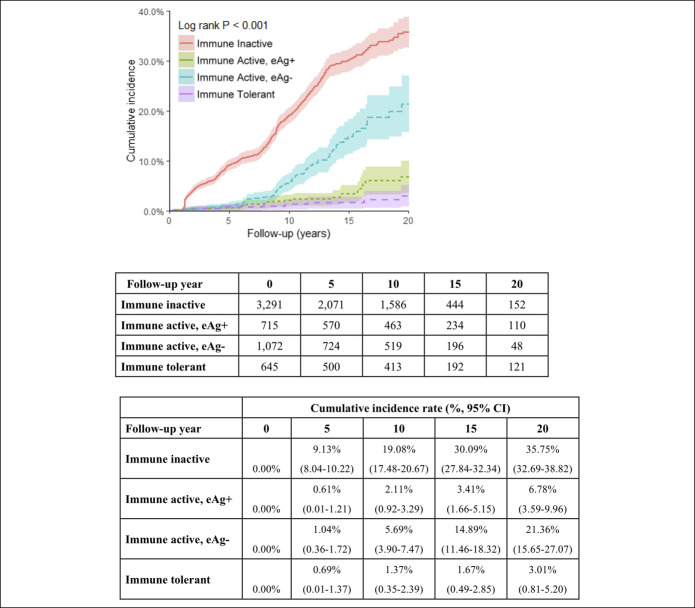
Cumulative incidence rate of spontaneous hepatitis B surface antigen seroclearance according to phases of chronic hepatitis B infection. Levels of significance: *P* < 0.001 (log-rank test). Color shades denote 95% CI of the cumulative incidence rate. The phase of infection was defined according to the American Association for the Study of Liver Diseases 2018 guideline. 95% CI, 95% confidence interval.

HBV genotype C patients had higher 20-year cumulative incidence rates than those with genotype B (27.34% [95% CI: 23.38–31.31] vs 18.55% [95% CI: 16.35–20.75], *P* < 0.001) (Figure 3D and see Table 6D, Supplementary Digital Content 1, http://links.lww.com/CTG/A366). Further stratification by age, sex, qHBsAg, and infection phase is shown in Figure 4 (see Supplementary Digital Content 1, http://links.lww.com/CTG/A366).

### Subgroup analysis by infection phases

Figure 4 shows the cumulative incidence rates according to phases of CHB and HBeAg status for immune active cases. Immune inactive patients had the highest cumulative incidence of spontaneous HBsAg seroclearance (35.75% [95% CI: 32.69–38.82]), followed by immune active, HBeAg-negative patients (21.36% [95% CI: 15.65–27.07]), immune active, HBeAg-positive patients (6.78% [95% CI: 3.59–9.96]), and immune-tolerant patients (3.01% [95% CI: 0.81–5.20]) (*P* < 0.001). When categorized by infection phase, the differences among the subgroups by age, sex, and qHBsAg level were consistently significant in patients with immune inactive phase and less so in the other 2 groups likely related to the low event rates in these 2 groups (see Figure 5A–C, Supplementary Digital Content 1, http://links.lww.com/CTG/A366).

### Predictors of spontaneous HBsAg seroclearance

In multivariable analysis (Table 3), being male (adjusted hazard ratio [aHR] = 1.66, 95% CI: 1.32–2.09), older (aHR = 1.16, 95% CI: 1.02–1.33 for 41–55 years; aHR = 1.21, 95% CI: 1.03–1.42 for >55 years), and HBeAg negative at baseline (aHR = 6.34, 95% CI: 4.23–9.52) were associated with a higher likelihood of attaining HBsAg seroclearance. The C statistic for the model is 0.995. The E values for variables with significance ranged from 1.59 to 12.16.

**Table 3.**
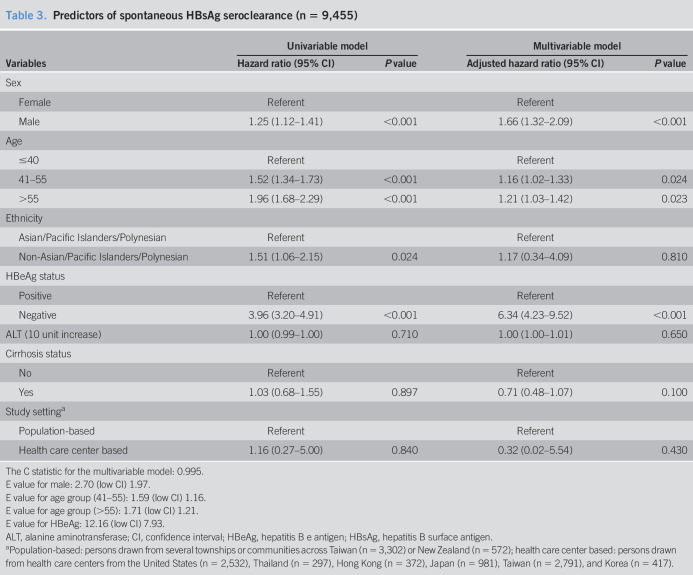
Predictors of spontaneous HBsAg seroclearance (n = 9,455)

Variables	Univariable model		Multivariable model	
	Hazard ratio (95% CI)	*P* value	Adjusted hazard ratio (95% CI)	*P* value
Sex				
Female	Referent		Referent	
Male	1.25 (1.12–1.41)	<0.001	1.66 (1.32–2.09)	<0.001
Age				
≤40	Referent		Referent	
41–55	1.52 (1.34–1.73)	<0.001	1.16 (1.02–1.33)	0.024
>55	1.96 (1.68–2.29)	<0.001	1.21 (1.03–1.42)	0.023
Ethnicity				
Asian/Pacific Islanders/Polynesian	Referent		Referent	
Non-Asian/Pacific Islanders/Polynesian	1.51 (1.06–2.15)	0.024	1.17 (0.34–4.09)	0.810
HBeAg status				
Positive	Referent		Referent	
Negative	3.96 (3.20–4.91)	<0.001	6.34 (4.23–9.52)	<0.001
ALT (10 unit increase)	1.00 (0.99–1.00)	0.710	1.00 (1.00–1.01)	0.650
Cirrhosis status				
No	Referent		Referent	
Yes	1.03 (0.68–1.55)	0.897	0.71 (0.48–1.07)	0.100
Study setting^a^				
Population-based	Referent		Referent	
Health care center based	1.16 (0.27–5.00)	0.840	0.32 (0.02–5.54)	0.430

The C statistic for the multivariable model: 0.995.

E value for male: 2.70 (low CI) 1.97.

E value for age group (41–55): 1.59 (low CI) 1.16.

E value for age group (>55): 1.71 (low CI) 1.21.

E value for HBeAg: 12.16 (low CI) 7.93.

ALT, alanine aminotransferase; CI, confidence interval; HBeAg, hepatitis B e antigen; HBsAg, hepatitis B surface antigen.

a Population-based: persons drawn from several townships or communities across Taiwan (n = 3,302) or New Zealand (n = 572); health care center based: persons drawn from health care centers from the United States (n = 2,532), Thailand (n = 297), Hong Kong (n = 372), Japan (n = 981), Taiwan (n = 2,791), and Korea (n = 417).

In separate models adjusting for sex, age, study setting, ethnicity, baseline HBeAg status, cirrhosis, and alanine aminotransferase level, higher HBV DNA level (>20,000 IU/mL: aHR = 0.35, 95% CI: 0.29–0.43; 2,000–20,000 IU/mL: aHR = 0.43, 95% CI: 0.35–0.51) (see Table 7A, Supplementary Digital Content 1, http://links.lww.com/CTG/A366) and higher qHBsAg level (≥1,000 IU/mL: aHR = 0.17, 95% CI: 0.14–0.20; 500–999 IU/mL: aHR = 0.32, 95% CI: 0.26–0.40) (see Table 7B, Supplementary Digital Content 1, http://links.lww.com/CTG/A366) were associated with significantly lower probability of seroclearance.

To further assess the association between infection phase and spontaneous HBsAg seroclearance, we adjusted for sex, age, ethnicity, and study setting and showed that immune active, eAg+, immune active, eAg−, and immune tolerant were significantly associated with lower probability of spontaneous HBsAg seroclearance (see Table 8, Supplementary Digital Content 1, http://links.lww.com/CTG/A366). Table 9 (see Supplementary Digital Content 1, http://links.lww.com/CTG/A366) demonstrates that genotype C was independently associated with higher probability of spontaneous HBsAg seroclearance (1.71 [95% CI: 1.44–2.04], *P* < 0.001) after controlling for sex, age, study setting, baseline HBeAg, alanine aminotransferase level, and HBV DNA.

## DISCUSSION

In this large, multinational cohort study that included 11,264 untreated patients with CHB, the annual rate of spontaneous HBsAg seroclearance was 1.31%. However, both males and females aged ≥55 years with immune inactive phase and qHBsAg <100 IU/mL presented an annual incidence rate higher than 7%. Overall, the 10- and 20-year cumulative incidence rates were 10.72% and 24.79%. On multivariable Cox regression, independent predictors of spontaneous seroclearance were male, 40 years or older, HBeAg negative, has genotype C infection compared with genotype B, and low levels of HBV DNA and qHBsAg. Compared with immune-inactive patients, immune-active (HBeAg-negative), immune-active (HBeAg-positive), and immune-tolerant patients were about 45%, 75%, and 90% less likely to experience spontaneous HBsAg seroclearance, respectively.

Our findings are unique for our large and diverse study population and the long duration of follow-up. Our recent meta-analysis of HBsAg seroclearance in both treated and untreated patients reported that the pooled annual rate of HBsAg seroclearance was 1.02%, and the cumulative incidence rates were 4.03% at 5 years, 8.16% at 10 years, and 17.99% at 15 years (13). Categorized by treatment history, the annual incidence rates in treated and untreated patients were 0.82% and 1.31%. In this study, using individual patient data of untreated patients with HBV, we were able to fill in these gaps and provide data on spontaneous HBsAg seroclearance rates for a comprehensive range of relevant clinical subgroups after controlling for key confounders. As a result, although there was no difference in sex and HBV genotype in our earlier meta-analysis, males and genotype C patients were found in the current study to be 70% and 80% more likely to achieve spontaneous HBsAg seroclearance compared with females and genotype B patients, respectively. On the other hand, while consistent with finding in our recent meta-analysis, which reported higher chance of spontaneous HBsAg seroclearance for HBeAg-negative compared to HBeAg-positive patients, the adjusted hazard ratio for spontaneous HBsAg seroclearance in the current study for HBeAg-negative patients was 6.34 (compared with only 1.74 times from unadjusted meta-analytic results of mixed treated and untreated patients). The current study also showed that the immune active phase was associated with lower HBsAg seroclearance compared with the immune inactive phase, and this is likely the reason for the lack of difference in HBsAg seroclearance rates between treated and untreated patients seen in the recent meta-analysis because treated patients were most likely immune-active patients, and untreated patients were more likely to be immune inactive.

The finding that HBV viral markers, such as lower HBV DNA and qHBsAg, confer a higher probability of spontaneous HBsAg seroclearance in a dose-dependent manner was supported by previous literature (10,17,18). This association was also manifested in our subgroup analysis stratified by infection phase, sex, age, qHBsAg level, and multivariable Cox regression.

Observations regarding the association between genotype and HBsAg seroclearance have been conflicting. One prior case-control study showed that patients with CHB with genotype B infection were more likely to achieve HBsAg seroclearance than those with genotype C infection (19). However, that study included less than 50 patients in each genotype subgroup. A larger study with longitudinal follow-up indicated a nonsignificant association between genotype and HBsAg seroclearance in patients with negative HBeAg (20). Another cohort study by Tseng et al. showed that HBeAg-negative patients with genotype C patients had a higher lifetime cumulative incidence of HBsAg seroclearance (21). In the current study with 3,934 patients with genotype B infection and 2,025 with genotype C, after adjusting for relevant demographic and clinical variables, genotype C was independently and significantly associated with a higher probability of achieving a spontaneous HBsAg seroclearance rate than genotype B. Previous report has indicated differences in geographic distribution and clinical outcomes of genotype C subtype B1 and B2 (formerly Ba and Bj) (22). B1 subtype was associated with a higher rate of basal core promoter mutation and a higher risk of developing advanced liver disease at younger age (22,23). However, we did not have subgenotype data to include in this study.

This study has several strengths. To our knowledge, this is the largest study on the incidence rates of spontaneous HBsAg seroclearance using individual patient-level data with long-term follow-up that also included both health care center and population-based data and from both North America and Asia Pacific. The large sample size and comprehensive set of variables enabled detailed subgroup analyses providing HBsAg seroclearance data for a whole host of specific relevant subgroups.

This study also has limitations. First, there were missing data on variables such as quantitative HBsAg and HBV DNA, precluding the inclusion of these variables into the main multivariable model. However, we did evaluate additional models using the same variables as in the main predictor model, plus these variables in the subsets of patients with available data (8,273 patients for HBV DNA and 7,011 for qHBsAg). Second, the study population may not represent the general HBV population, as two-thirds of the patients were from health care centers. To overcome this limitation, we performed separate analysis for health care–based and population-based cohorts, which showed higher rates of HBsAg seroclearance in the latter. In addition, our findings may not be generalizable to patients infected with genotypes other than B and C due to the small sample size. This study also included very few non-Asian/Pacific Islanders/Polynesians (1.9%). However, the majority of CHB cases in the world are Asian/Pacific Islanders/Polynesians. Third, the number of patients with immune-active HBeAg-positive CHB and immune-tolerant CHB was small, limiting further subgroup analyses in these 2 subgroups. Fourth, the seroclearance rates derived from certain subgroup such as immune inactive carriers may not be helpful in planning trials, which aim at patients with untreated immune active disease. However, the findings are helpful in understanding the natural history of HBV infection. Finally, follow-up testing after confirmation of HBsAg seroclearance was limited, and we could not calculate the rate of HBsAg seroreversion. Thus, we could not address the durability of HBsAg seroclearance.

Using patient-level data from a large cohort of 11,264 untreated patients with CHB from 12 study sites in 7 countries/regions, we found that the overall annual spontaneous HBsAg seroclearance rate was low at 1.31%. Over 20 years, the cumulative HBsAg seroclearance rate increased to 25%, with rates ranging from 3.01% to 60.25% in different subgroups, highlighting the need to consider patient demographics and clinical phenotype in the management of patients with CHB and in planning future HBV cure clinical trials.

## CONFLICTS OF INTEREST

**Guarantor of the article:** Mindie H. Nguyen, MD, MAS.

**Specific author contributions:** Y.H.Y. and M.H.N. designed the study. All authors performed data collection and/or interpretation. Y.H.Y., H.J.H., D.J., A.S.L., and M.H.N. performed data analysis. Y.H.Y. and M.H.N. drafted the manuscript. All authors provided critical review and/or revision and approved the final draft submitted.

**Financial support:** None to report.

**Potential competing interests:** T.-C. Tseng: research support: the Ministry of Science and Technology, Executive Yuan, Taiwan (MOST 105-2314-B-303-008), and National Taiwan University Hospital, Taipei, Taiwan (NTUH 106-003626); speaker: AbbVie, Bristol-Myers Squibb, and Gilead Sciences. H. N. Trinh: research support: Gilead; consultancy/advisory board: Gilead; speaker: Gilead; stocks: Gilead. J. Y. Y. Fung: research support: Novartis. T. Ungtrakul: research support: AstraZeneca; speaker: AstraZeneca. M.-L. Yu: research support: AbbVie, BMS, Gilead, Torpedo, and Merck; consultant: AbbVie, Abbott, Ascletis, BMS, Gilead, Merck, and PharmaEssentia; speaker: AbbVie, Abbott, Ascletis, BMS, Gilead, and Merck. T. Tanwandee: research support: Roche, Merck, and ContraVir. E. Gane: consultancy/advisory board: AbbVie, Arrowhead, Assembly, Gilead Sciences, Janssen, Roche, VIR; Speakers, AbbVie, and Gilead Sciences. R. C. Cheung: research support: Gilead Sciences. M.-F. Yuen: research support and/or consultancy/advisory board: AbbVie, Arrowhead Pharmaceuticals, Biocartis, Bristol-Myers Squibb, Fujirebio, Gilead Sciences, GlaxoSmithKline, LF Asia Limited, Merck Sharp & Dohme, Novartis Pharmaceuticals, Roche, and Sysmex Corporation. F. Suzuki: speaker: Bristol-Myers Squibb. J.-H. Kao: consultancy/advisory board: AbbVie, BMS, Gilead Sciences, and MSD; speaker, AbbVie, Ascletis, BMS, Gilead Sciences, and MSD. A. S. Lok: research support: Assembly Biosciences, Bristol-Myers Squibb, Gilead, and TARGET Pharma; consultancy/advisory board: CLEAR-B, DiaSorin, Epigenomics, Gilead, Huahui, Roche, Spring Banks, TARGET Pharma, and Viravaxx. H.-I. Yang: research support: Academia Sinica. M. H. Nguyen: research support: Pfizer, Gilead Sciences, Janssen, National Cancer Institute, and B.K.Kee Foundation; consulting/advisory board: Alnylam, Janssen, Intercept, Spring Banks, Bayer, Gilead, Dynavax, Eisai, Exact Sciences, and Novartis. Y. H. Yeo, H. J. Ho, T. Hosaka, C. Cunningham, M.-S. Kwak, J. Li, J. Zhang, A. K. Le, L. Henry, D. Jeong, T. Sriprayoon, H.-S. Lee, M. Kobayashi, and C.-Y. Wu: none to report.

**Study Highlights**

**WHAT IS KNOWN**

✓ Spontaneous HBsAg seroclearance is a key milestone of the natural history of HBV infection.
✓ Previous studies are limited by the insufficient sample size and/or follow-up, and recent meta-analyses are limited by study-level data and lack of adjustment for confounders.

**WHAT IS NEW HERE**

✓ This multinational cohort study analyzes 11,264 untreated patients with CHB (106,192 person-years) and shows that the annual and 20-year cumulative incidence rates of spontaneous HBsAg seroclearance are 1.31% (95% CI: 1.25–1.38) and 32.26% (95% CI: 29.59–34.94), respectively.
✓ The seroclearance rates are categorized by infection phase, with further stratification by HBeAg status, sex, age, and qHBsAg.
✓ On multivariable Cox regression, male, older age, negative HBeAg, and genotype C predict higher seroclearance rates, as do lower HBV DNA, lower qHBsAg, and inactive carrier state.

**TRANSLATIONAL IMPACT**

✓ Understanding of spontaneous HBsAg seroclearance in relevant patient subgroups has key implications in planning of future trials and the management of patients with CHB.

## Supplementary Material

SUPPLEMENTARY MATERIAL
