# Supplementary material for: Incidence, Factors, and Patient-Level Data for Spontaneous HBsAg Seroclearance: A Cohort Study of 11,264 Patients
Source: Clin Transl Gastroenterol. 2020 Sep 15;11(9):e00196. doi: 10.14309/ctg.0000000000000196 (PMC7494149; doi:10.14309/ctg.0000000000000196)
Supplement: SUPPLEMENTARY MATERIAL [file ct9-11-e00196-s001.docx]

**Incidence, factors, and patient-level data for spontaneous HBsAg seroclearance: A multinational cohort study of 11,264 patients**

Yee Hui Yeo, Tai-Chung Tseng, Tetsuya Hosaka, Chris Cunningham, James Yan Yue Fung, Hsiu J. Ho, Min-Sun Kwak, Huy N. Trinh, Teerapat Ungtrakul, Ming-Lung Yu, Mariko Kobayashi, An K. Le, Linda Henry, Jiayi Li, Jian Zhang, Tassanee Sriprayoon, Donghak Jeong, Tawesak Tanwandee, Ed Gane, Ramsey C. Cheung, Chun-Ying Wu, Anna S. Lok, Hyo-Suk Lee, Fumitaka Suzuki, Man-Fung Yuen, Jia-Horng Kao, Hwai-I Yang, Mindie H. Nguyen

**Table of Content**

**Supplementary Table 1. The definition of phases of chronic hepatitis B infection. (Page 3)**

**Supplementary Table 2.** **Annual incidence rate of spontaneous HBsAg seroclearance, overall and in subgroups. (Page 4)**

**Supplementary Table 3. Annual incidence rate of spontaneous HBsAg seroclearance by infection phase, sex, and age (Page 5)**

**Supplementary Table 4. Number of patients in each phase of chronic HBV infection, stratified by sex, age, and qHBsAg level. (Page 7)**

**Supplementary Table 5. Number of patients at risk and cumulative incidence rate of spontaneous HBsAg seroclearance, stratified by: (A) age (B) sex, (C) study setting, and (D) ethnicity. (Page 10)**

**Supplementary Table 6. Patients at risk and cumulative incidence rate of spontaneous HBsAg seroclearance, stratified by (A) HBeAg status, (B) HBV DNA, (C) qHBsAg, (D) genotype. (Page 12)**

**Supplementary Table 7. Additional Cox regression models. (A) Main regression model + HBV DNA (n=8,273). (B) Main regression model + qHBsAg (n=7,011). (Page 14)**

**Supplementary Table 8. The association between infection phase and spontaneous HBsAg seroclearance (n=5,553). (Page 15)**

**Supplementary Table 9. The association between HBV genotype and spontaneous HBsAg seroclearance. (n=5,581). (Page 16)**

**Supplementary Figure 1. Cumulative incidence rates of spontaneous HBsAg seroclearance by study setting, stratified by** **age. (Page 21)**

**Supplementary Figure 2. Cumulative incidence rates of spontaneous HBsAg seroclearance by sex and by age. (Page 15)**

**Supplementary Figure 3. Cumulative incidence rates of spontaneous HBsAg seroclearance by HBeAg status, stratified by: (A) age, (B) sex, (C) HBV DNA, (D) qHBsAg, and (E) genotype. (Page 16)**

**Supplementary Figure 4. Cumulative incidence rates of spontaneous HBsAg seroclearance rate by genotype, stratified by: (A) age, (B) sex, (C) qHBsAg, and (D) infection phase. (Page 22)**

**Supplementary Figure 5. Cumulative incidence rates of spontaneous HBsAg seroclearance by infection phase, stratified by: (A) age, (B) sex, and (C) qHBsAg. (Page 27)**

**Supplementary Table 1. The definition of phases of chronic hepatitis B infection**

| **Phase** | **HBeAg status** | **HBV DNA** | **ALT Level*** |
| --- | --- | --- | --- |
| **Immune tolerant** | HBeAg+ | > 1,000,000 IU/ml | Normal |
| **Immune active, eAg+** | HBeAg+ | > 2,0000 IU/mL | Elevated |
| **Immune active, eAg-** | HBeAg- | > 2,000 IU/mL | Elevated |
| **Immune inactive** | HBeAg- | < 2,000 IU/mL | Normal |

ALT, alanine aminotransferase

*Upper limit of normal: 35 U/mL for males; 25 U/mL for females

**Supplementary Table 2.** **Annual incidence rate of spontaneous HBsAg seroclearance, overall and in subgroups**

|  | **Patients** | **HBsAg seroclearance** | **Person-years** | **Annual incidence rate (%, 95% CI)** |
| --- | --- | --- | --- | --- |
| **Overall** | **11,264** | **1,393** | **106,192.00** | **1.31 (1.25-1.38)** |
| **Sex** |  |  |  |  |
| Female | 4,489 | 418 | 39,848.48 | 1.05 (0.95-1.15) |
| Male | 6,775 | 975 | 66,343.53 | 1.47 (1.38-1.57) |
| **Age** |  |  |  |  |
| <=40 | 5,182 | 587 | 57,768.10 | 1.02 (0.94-1.10) |
| 41-55 | 4,140 | 541 | 35,039.41 | 1.54 (1.42-1.68) |
| >55 | 1,942 | 265 | 13,384.50 | 1.98 (1.75-2.23) |
| **HBeAg status** |  |  |  |  |
| Negative | 8,710 | 1,252 | 77,489.87 | 1.62 (1.53-1.71) |
| Positive | 1,908 | 92 | 22,358.16 | 0.41 (0.33-0.51) |
| **Cirrhosis status** |  |  |  |  |
| No | 10,367 | 1,223 | 91,515.22 | 1.34 (1.26-1.41) |
| Yes | 325 | 25 | 1,727.56 | 1.45 (0.96-2.16) |
| **Study setting** |  |  |  |  |
| Population-based | 3,874 | 713 | 39,595.35 | 1.80 (1.67-1.94) |
| Healthcare center-based | 7,390 | 680 | 66,596.66 | 1.02 (0.95-1.10) |
| **Ethnicity** |  |  |  |  |
| Asian/Pacific Islanders/Polynesian | 10,899 | 1,351 | 102,525.63 | 1.32 (1.25-1.39) |
| Non-Asian/Pacific Islanders/Polynesian | 206 | 41 | 2,970.12 | 1.38 (1.01-1.89) |
| **HBV DNA (IU/mL)** |  |  |  |  |
| ≤2,000 | 4,489 | 779 | 37,764.56 | 2.06 (1.92-2.21) |
| 2,001-20,000 | 1,819 | 173 | 18,401.50 | 0.94 (0.81-1.09) |
| >20,000 | 3,058 | 203 | 34,924.37 | 0.58 (0.51-0.67) |
| **Quantitative HBsAg (IU/mL)** |  |  |  |  |
| <100 | 1,445 | 611 | 12018.33 | 5.08 (4.70-5.50) |
| 100-1,000 | 1,905 | 312 | 20275.91 | 1.54 (1.38-1.72) |
| >1,000 | 4,192 | 306 | 52831.21 | 0.58 (0.52-0.65) |
| **Genotype** |  |  |  |  |
| B | 3,934 | 394 | 45,390.52 | 0.87 (0.79-0.96) |
| C | 2,025 | 265 | 19,051.56 | 1.39 (1.23-1.57) |

**Supplementary Table 3. Annual incidence rate of spontaneous HBsAg seroclearance by infection phase, sex, and age**

| **Infection phase** | **Sex** | **Age group** | **N** | **Event** | **Follow-up  (Person-years)** | **Annual Incidence Rate (%) (95%CI)*** |
| --- | --- | --- | --- | --- | --- | --- |
| **Immune inactive** | **Overall** | | 3,291 | 647 | 29179.64 | 2.22% (2.05%-2.39%) |
|  | **Female** | **<=40** | 555 | 75 | 5240.26 | 1.43% (1.14%-1.80%) |
|  |  | **41-50** | 403 | 50 | 3400.98 | 1.47% (1.10%-1.95%) |
|  |  | **>50** | 426 | 71 | 2821.697 | 2.52% (1.98%-3.18%) |
|  | **Male** | **<=40** | 733 | 141 | 7506.20 | 1.88% (1.59%-2.22%) |
|  |  | **41-50** | 522 | 120 | 4948.58 | 2.42% (2.02%-2.90%) |
|  |  | **>50** | 652 | 190 | 5261.92 | 3.61% (3.13%-4.16%) |
| **Immune active, eAg+** | **Overall** | | 715 | 33 | 8812.50 | 0.37% (0.26%-0.53%) |
|  | **Female** | **<=40** | 209 | 11 | 2892.79 | 0.38% (0.20%-0.70%) |
|  |  | **41-50** | 53 | 1 | - | - |
|  |  | **>50** | 38 | 3 | 300.79 | 1.00% (0.26%-3.13%) |
|  | **Male** | **<=40** | 286 | 14 | 3710.15 | 0.38% (0.21%-0.65%) |
|  |  | **40-55** | 80 | 1 | - | - |
|  |  | **>55** | 49 | 3 | 405.87 | 0.74% (0.19%-2.33%) |
| **Immune active, eAg-** | **Overall** | | 1,072 | 86 | 10002.79 | 0.86% (0.69%-1.07%) |
|  | **Female** | **<=40** | 114 | 7 | 1091.24 | 0.64% (0.28%-1.38%) |
|  |  | **40-55** | 123 | 9 | 1140.09 | 0.79% (0.39%-1.55%) |
|  |  | **>55** | 146 | 6 | 1159.00 | 0.52% (0.21%-1.18%) |
|  | **Male** | **<=40** | 270 | 28 | 2787.68 | 1.00% (0.68%-1.47%) |
|  |  | **40-55** | 219 | 23 | 2094.99 | 1.10% (0.71%-1.67%) |
|  |  | **>55** | 200 | 13 | 1729.78 | 0.75% (0.42%-1.32%) |
| **Immune tolerant** | **Overall** | | 645 | 18 | 8109.05 | 0.22% (0.14%-0.36%) |
|  | **Female** | **<=40** | 156 | 2 | 1933.18 | 0.10% (0.02%-0.42%) |
|  |  | **40-55** | 42 | 1 | - | - |
|  |  | **>55** | 30 | 1 | - | - |
|  | **Male** | **<=40** | 278 | 9 | 4260.91 | 0.21% (0.10%-0.42%) |
|  |  | **41-50** | 82 | 3 | 794.94 | 0.38% (0.10%-1.20%) |
|  |  | **>50** | 57 | 2 | 476.99 | 0.42% (0.07%-1.68%) |

*Data analyzed only for subgroups with at least 20 patients and at least 2 HBsAg seroclearance events

**Supplementary Table 4. Number of patients in each phase of chronic HBV infection, stratified by sex, age, and qHBsAg level.** *Data analyzed only for subgroups with at least 20 patients and at least 2 HBsAg seroclearance events

| **Infection phase** | **Sex** | **Age group** | **qHBsAg** | **Number of patients** | **HBsAg seroclearance** | **Follow-up (person- year)** | **Annual incidence Rate* (95% CI)** |
| --- | --- | --- | --- | --- | --- | --- | --- |
| **Immune inactive** | **Female** | **<=40** | **<100** | 106 | 40 | 816.30 | 4.90% (3.57-6.67) |
|  |  |  | **100-1000** | 142 | 19 | 1511.96 | 1.26% (0.78-1.99) |
|  |  |  | **>1000** | 231 | 12 | 2543.85 | 0.47% (0.26-0.85) |
|  |  | **41-55** | **<100** | 147 | 48 | 1082.44 | 4.43% (3.32-5.88) |
|  |  |  | **100-1000** | 139 | 17 | 1184.96 | 1.43% (0.87-2.34) |
|  |  |  | **>1000** | 174 | 8 | 1761.34 | 0.45% (0.21-0.93) |
|  |  | **>55** | **<100** | 80 | 30 | 393.72 | 7.62% (5.28-10.81) |
|  |  |  | **100-1000** | 61 | 8 | 514.63 | 1.55% (0.72-3.16) |
|  |  |  | **>1000** | 50 | 1 | - | - |
|  | **Male** | **<=40** | **<100** | 188 | 68 | 1684.40 | 4.04% (3.17-5.12) |
|  |  |  | **100-1000** | 152 | 36 | 1732.22 | 2.08% (1.48-2.90) |
|  |  |  | **>1000** | 306 | 33 | 3665.99 | 0.90% (0.63-1.28) |
|  |  | **41-55** | **<100** | 249 | 121 | 2231.93 | 5.42% (4.54-6.46) |
|  |  |  | **100-1000** | 189 | 38 | 2057.34 | 1.85% (1.33-2.55) |
|  |  |  | **>1000** | 191 | 17 | 2138.79 | 0.79% (0.48-1.30) |
|  |  | **>55** | **<100** | 176 | 99 | 1367.82 | 7.24% (5.95-8.77) |
|  |  |  | **100-1000** | 78 | 16 | 822.97 | 1.94% (1.15-3.21) |
|  |  |  | **>1000** | 61 | 1 | - | - |
| **Immune active, eAg+** | **Female** | **<=40** | **<100** | 0 | 0 | - | - |
|  |  |  | **100-1000** | 9 | 1 | - | - |
|  |  |  | **>1000** | 137 | 10 | 2347.34 | 0.43% (0.22-0.81) |
|  |  | **41-55** | **<100** | 0 | 0 | - | - |
|  |  |  | **100-1000** | 3 | 0 | - | - |
|  |  |  | **>1000** | 51 | 3 | 593.85 | 0.51% (0.13-1.60) |
|  |  | **>55** | **<100** | 1 | 0 | - | - |
|  |  |  | **100-1000** | 1 | 0 | - | - |
|  |  |  | **>1000** | 15 | 1 | - | - |
|  | **Male** | **<=40** | **<100** | 5 | 0 | - | - |
|  |  |  | **100-1000** | 11 | 1 | - | - |
|  |  |  | **>1000** | 206 | 12 | 3079.36 | 0.39% (0.21-0.70) |
|  |  | **41-55** | **<100** | 1 | 0 | - | - |
|  |  |  | **100-1000** | 9 | 0 | - | - |
|  |  |  | **>1000** | 63 | 2 | 803.55 | 0.25% (0.04-1.00) |
|  |  | **>55** | **<100** | 1 | 0 | - | - |
|  |  |  | **100-1000** | 5 | 0 | - | - |
|  |  |  | **>1000** | 12 | 1 | - | - |
| **Immune active, eAg-** | **Female** | **<=40** | **<100** | 1 | 0 | - | - |
|  |  |  | **100-1000** | 10 | 1 | - | - |
|  |  |  | **>1000** | 42 | 6 | 674.50 | 0.89% (0.36-2.03) |
|  |  | **41-55** | **<100** | 9 | 2 | 121.11 | 1.65% (0.29-6.43) |
|  |  |  | **100-1000** | 32 | 4 | 365.84 | 1.09% (0.35-2.97) |
|  |  |  | **>1000** | 58 | 5 | 766.60 | 0.65% (0.24-1.61) |
|  |  | **>55** | **<100** | 4 | 2 | 58.61 | 3.41% (0.59-12.83) |
|  |  |  | **100-1000** | 14 | 0 | - | - |
|  |  |  | **>1000** | 22 | 1 | - | - |
|  | **Male** | **<=40** | **<100** | 9 | 2 | 103.65 | 1.93% (0.33-7.48) |
|  |  |  | **100-1000** | 37 | 9 | 493.53 | 1.82% (0.89-3.56) |
|  |  |  | **>1000** | 116 | 14 | 1654.95 | 0.85% (0.48-1.45) |
|  |  | **41-55** | **<100** | 9 | 1 | - | - |
|  |  |  | **100-1000** | 65 | 20 | 788.38 | 2.54% (1.60-3.96) |
|  |  |  | **>1000** | 97 | 8 | 1241.81 | 0.64% (0.30-1.32) |
|  |  | **>55** | **<100** | 12 | 4 | 125.30 | 3.19% (1.03-8.47) |
|  |  |  | **100-1000** | 26 | 0 | - | - |
|  |  |  | **>1000** | 30 | 1 | - | - |
| **Immune tolerant** | **Female** | **<=40** | **<100** | 0 | 0 | - | - |
|  |  |  | **100-1000** | 4 | 0 | - | - |
|  |  |  | **>1000** | 129 | 2 | 1792.59 | 0.11% (0.02-0.45) |
|  |  | **41-55** | **<100** | 0 | 0 | - | - |
|  |  |  | **100-1000** | 4 | 0 | - | - |
|  |  |  | **>1000** | 44 | 1 | - | - |
|  |  | **>55** | **<100** | 0 | 0 | - | - |
|  |  |  | **100-1000** | 1 | 0 | - | - |
|  |  |  | **>1000** | 18 | 1 | - | - |
|  | **Male** | **<=40** | **<100** | 4 | 1 | - | - |
|  |  |  | **100-1000** | 7 | 0 | - | - |
|  |  |  | **>1000** | 248 | 8 | 3978.32 | 0.20% (0.09-0.41) |
|  |  | **41-55** | **<100** | 2 | 0 | - | - |
|  |  |  | **100-1000** | 10 | 0 | - | - |
|  |  |  | **>1000** | 81 | 5 | 844.39 | 0.59% (0.22-1.46) |
|  |  | **>55** | **<100** | 1 | 0 | - | - |
|  |  |  | **100-1000** | 1 | 0 | - | - |
|  |  |  | **>1000** | 24 | 0 | - | - |

**Supplementary Table 5. Number of patients at risk and cumulative incidence rate of spontaneous HBsAg seroclearance, stratified by: (A) age (B) sex, (C) study setting, and (D) ethnicity**

**(A) Age**

| **Year** | **0** | **5** | **10** | **15** | **20** |
| --- | --- | --- | --- | --- | --- |
| **Patients (n)** |  |  |  |  |  |
| <=40 | 5,182 | 3,608 | 2,899 | 1,437 | 757 |
| 41-55 | 4,140 | 2,521 | 1,823 | 563 | 190 |
| >55 | 1,942 | 1,002 | 615 | 165 | 30 |
| **Cumulative incidence rate (%) (95% CI)** | | |  |  |  |
| <=40 | 0.00% | 3.41%  (2.86-3.95) | 7.31%  (6.48-8.14) | 13.14% (11.89-14.39) | 19.69% (17.89-21.49) |
| 41-55 | 0.00% | 5.42%  (4.63-6.21) | 12.87%  (11.58-14.16) | 23.36%  (21.29-25.43) | 29.13%  (26.18-32.09) |
| >55 | 0.00% | 7.21%  (5.86-8.56) | 17.41%  (15.07-19.75) | 30.35%  (26.64-34.05) | 33.19%  (28.64-37.75) |

**(B) Sex**

| **Year** | **0** | **5** | **10** | **15** | **20** |
| --- | --- | --- | --- | --- | --- |
| **Patients (n)** |  |  |  |  |  |
| Female | 4,489 | 2,565 | 1,915 | 889 | 390 |
| Male | 6,775 | 4,566 | 3,422 | 1,276 | 587 |
| **Cumulative incidence rate (%) (95% CI)** | | |  |  |  |
| Female | 0.00% | 4.29%  (3.60-4.97) | 8.56%  (7.51-9.61) | 15.09%  (13.48-16.70) | 19.48%  (17.32-21.64) |
| Male | 0.00% | 4.98%  (4.41-5.55) | 11.89%  (10.96-12.81) | 20.88%  (19.49-22.28) | 27.90%  (25.93-29.86) |

**(C) Study setting**

| **Year** | **0** | **5** | **10** | **15** | **20** |
| --- | --- | --- | --- | --- | --- |
| **Patients (n)** |  |  |  |  |  |
| Population-based | 3,874 | 2,724 | 2,165 | 499 | 408 |
| Healthcare center-based | 7,390 | 4,407 | 3,172 | 1,666 | 569 |
| **Cumulative incidence rate (%) (95% CI)** | | |  |  |  |
| Population-based | 0.00% | 6.24%  (5.42-7.07) | 14.36%  (13.09-15.63) | 27.02%  (24.80-29.25) | 32.26%  (29.59-34.94) |
| Healthcare center-based | 0.00% | 3.90%  (3.39-4.41) | 8.31%  (7.51-9.12) | 14.16%  (12.99-15.34) | 20.70%  (18.89-22.52) |

**(D) Ethnicity**

| **Year** | **0** | **5** | **10** | **15** | **20** |
| --- | --- | --- | --- | --- | --- |
| **Patients (n)** |  |  |  |  |  |
| Asian/Pacific Islanders/Polynesian | 10,899 | 6,944 | 5,194 | 2,066 | 898 |
| Non-Asian/Pacific Islanders/Polynesian | 206 | 138 | 121 | 93 | 78 |
| **Cumulative incidence rate (%) (95% CI)** | | |  |  |  |
| Asian/Pacific Islanders/Polynesian | 0.00% | 4.86%  (4.41-5.32) | 10.96%  (10.24-11.68) | 18.90%  (17.82-19.99) | 24.98%  (23.46-26.49) |
| Non-Asian/Pacific Islanders/Polynesian | 0.00% | 0.56%  (0.00-1.65) | 1.34%  (0.00-3.22) | 15.13%  (8.62-21.65) | 20.90%  (13.35-28.45) |

**Supplementary Table 6. Patients at risk and cumulative incidence rate of spontaneous HBsAg seroclearance, stratified by (A) HBeAg status, (B) HBV DNA, (C) qHBsAg, (D) genotype**

**(A) HBeAg status**

| **Year** | **0** | **5** | **10** | **15** | **20** |
| --- | --- | --- | --- | --- | --- |
| **Patients (n)** |  |  |  |  |  |
| HBeAg- | 8,710 | 5,317 | 3,944 | 1,420 | 591 |
| HBeAg+ | 1,908 | 1,382 | 1,090 | 571 | 329 |
| **Cumulative incidence rate (%) (95% CI)** | | |  |  |  |
| HBeAg- | 0.00% | 5.80%  (5.24-6.36) | 13.09%  (12.20-13.97) | 23.14%  (21.79-24.48) | 30.25%  (28.36-32.15) |
| HBeAg+ | 0.00% | 1.22%  (0.69-1.75) | 2.82%  (1.95-3.70) | 4.27%  (3.05-5.50) | 7.31%  (5.33-9.28) |

**(B) HBV DNA (IU/mL)**

| **Year** | **0** | **5** | **10** | **15** | **20** |
| --- | --- | --- | --- | --- | --- |
| **Patients (n)** |  |  |  |  |  |
| ≤2,000 | 4,489 | 2,685 | 1,964 | 577 | 199 |
| 2,001-20,000 | 1,819 | 1,219 | 958 | 387 | 183 |
| >20,000 | 3,058 | 2,251 | 1,816 | 841 | 426 |
| **Cumulative incidence rate (%) (95% CI)** | | |  |  |  |
| ≤2,000 | 0.00% | 8.14%  (7.23-9.04) | 17.97%  (16.57-19.36) | 29.00%  (26.99-31.01) | 34.76%  (31.99-37.52) |
| 2,001-20,000 | 0.00% | 1.02%  (0.48-1.55) | 4.90%  (3.65-6.14) | 13.59%  (11.14-16.04) | 22.29%  (18.49-26.08) |
| >20,000 | 0.00% | 0.89%  (0.53-1.26) | 3.28%  (2.53-4.03) | 7.71%  (6.36-9.06) | 11.82%  (9.80-13.83) |

**(C) qHBsAg (IU/mL)**

| **Year** | **0** | **5** | **10** | **15** | **20** |
| --- | --- | --- | --- | --- | --- |
| **Patients (n)** |  |  |  |  |  |
| <100 | 1,445 | 888 | 627 | 152 | 48 |
| 100-1,000 | 1,905 | 1,454 | 1,181 | 394 | 121 |
| >1,000 | 4,192 | 3,348 | 2,876 | 1,290 | 683 |
| **Cumulative incidence rate (%) (95% CI)** | | |  |  |  |
| <100 | 0.00% | 22.48% (20.18-24.78) | 39.12%  (36.31-41.93) | 55.76%  (52.33-59.19) | 60.25%  (56.05-64.46) |
| 100-1,000 | 0.00% | 2.79%  (1.98-3.59) | 10.60%  (9.02-12.18) | 24.09%  (21.31-26.86) | 33.82%  (29.53-38.11) |
| >1,000 | 0.00% | 0.68%  (0.41-0.95) | 2.83%  (2.26-3.40) | 6.64%  (5.63-7.64) | 12.00%  (10.33-13.67) |

**(D) Genotype**

| **Year** | **0** | **5** | **10** | **15** | **20** |
| --- | --- | --- | --- | --- | --- |
| **Patients (n)** |  |  |  |  |  |
| B | 3,934 | 3,112 | 2,673 | 1,053 | 347 |
| C | 2,025 | 1,399 | 1,040 | 303 | 94 |
| **Cumulative incidence rate (%) (95% CI)** | | |  |  |  |
| B | 0.00% | 2.24%  (1.74-2.74) | 6.20%  (5.36-7.05) | 12.93%  (11.54-14.32) | 18.55%  (16.35-20.75) |
| C | 0.00% | 4.52%  (3.53-5.51) | 11.67%  (10.00-13.33) | 20.42%  (17.77-23.07) | 27.34%  (23.38-31.31) |

**Supplementary Table 7a. Main regression model + HBV DNA (n=8,273)**

|  | **Univariable model** |  | **Multivariable model** |  |
| --- | --- | --- | --- | --- |
| **HBV DNA level (IU/mL)** | **Hazard ratio  (95% CI)** | ***P*-value** | **Adjusted hazard ratio* (95% CI)** | ***P*-value** |
| <2,000 | Referent |  | Referent |  |
| 2,001-20,000 | 0.46 (0.39-0.55) | <0.001 | 0.43 (0.35-0.51) | <0.001 |
| >20,000 | 0.28 (0.24-0.33) | <0.001 | 0.35 (0.29-0.43) | <0.001 |

*Adjusted for sex, age, baseline HBeAg, cirrhosis, ethnicity, study setting, and ALT level

**Supplementary Table 7b. Main regression model + qHBsAg (n=7,011)**

|  | **Univariable model** |  | **Multivariable model** |  |
| --- | --- | --- | --- | --- |
| **Quantitative HBsAg (IU/mL)** | **Hazard ratio  (95% CI)** | ***P*-value** | **Adjusted hazard ratio* (95% CI)** | ***P*-value** |
| < 100 | Referent |  | Referent |  |
| 100-1,000 | 0.35 (0.29-0.43) | <0.001 | 0.32 (0.26-0.40) | <0.001 |
| > 1,000 | 0.14 (0.12-0.17) | <0.001 | 0.17 (0.14-0.20) | <0.001 |

*Adjusted for sex, age, baseline HBeAg, cirrhosis status, ethnicity, study setting, and ALT level

**Supplementary Table 8. The association between infection phase and spontaneous HBsAg seroclearance (n=5,553)**

|  | **Univariable model** |  | **Multivariable model** |  |
| --- | --- | --- | --- | --- |
| **Phase of infection** | **Hazard ratio (95% CI)** | ***P*-value** | **Adjusted hazard ratio* (95% CI)** | ***P*-value** |
| Immune inactive | Referent |  | Referent |  |
| Immune active, eAg+ | 0.21 (0.15-0.30) | <0.001 | 0.239 (0.17-0.34) | <0.001 |
| Immune active, eAg- | 0.578 (0.46-0.73) | <0.001 | 0.547 (0.43-0.69) | <0.001 |
| Immune tolerant | 0.093 (0.06-0.15) | <0.001 | 0.107 (0.07-0.17) | <0.001 |

*Adjusted for sex, age, ethnicity, and study setting

**Supplementary Table 9. The association between HBV genotype and spontaneous HBsAg seroclearance**

**(n=5,581)**

|  | **Univariable model** |  | **Multivariable model** |  |
| --- | --- | --- | --- | --- |
| **HBV genotype** | **Hazard ratio (95% CI)** | ***P*-value** | **Adjusted hazard ratio* (95% CI)** | ***P*-value** |
| B | Referent |  | Referent |  |
| C | 1.49 (1.25-1.76) | <0.001 | 1.71 (1.44-2.04) | <0.001 |

*Adjusted for sex, age, study setting, baseline HBeAg, ALT level, and HBV DNA

**Supplementary Figure 1. Cumulative incidence rates of spontaneous HBsAg seroclearance by study setting, stratified by** **age**

**
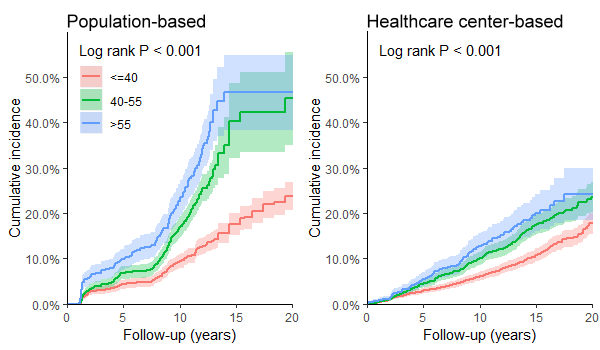
**

| **Year** | **0** | **5** | **10** | **15** | **20** |
| --- | --- | --- | --- | --- | --- |
| **Patients (n)** | |  |  |  |  |
| **Population-based** |  |  |  |  |  |
| <=40 | 1,681 | 1,301 | 1,121 | 442 | 384 |
| 41-55 | 1,454 | 992 | 754 | 33 | 17 |
| >55 | 739 | 431 | 290 | 24 | 7 |
| **Healthcare center-based** | |  |  |  |  |
| <=40 | 3,501 | 2,307 | 1,778 | 995 | 373 |
| 41-55 | 2,686 | 1,529 | 1,069 | 530 | 173 |
| >55 | 1,203 | 571 | 325 | 141 | 23 |
| **Cumulative incidence rate (%) (95% CI)** | | |  |  |  |
| **Population-based** |  |  |  |  |  |
| <=40 | 0.00% | 4.28% (3.26-5.31) | 9.40% (7.86-10.94) | 17.52% (15.06-19.97) | 23.67% (20.61-26.74) |
| 41-55 | 0.00% | 6.89% (5.46-8.33) | 16.87% (14.61-19.14) | 40.31% (32.00-48.63) | 45.22% (34.94-55.51) |
| >55 | 0.00% | 9.78% (7.40-12.15) | 22.88% (19.10-26.67) | 46.51% (38.19-54.82) | 46.51% (38.19-54.82) |
| **Healthcare center-based** | |  |  |  |  |
| <=40 | 0.00% | 2.97% (2.34-3.61) | 6.09% (5.13-7.05) | 10.78% (9.36-12.20) | 17.78% (15.45-20.10) |
| 41-55 | 0.00% | 4.57% (3.64-5.50) | 10.03% (8.54-11.53) | 17.38% (15.18-19.58) | 23.56% (20.33-26.79) |
| >55 | 0.00% | 5.48% (3.89-7.07) | 12.72% (9.93-15.51) | 20.05% (15.98-24.13) | 24.21% (18.52-29.89) |

**Supplementary Figure 2. Cumulative incidence rates of spontaneous HBsAg seroclearance by sex and by age**


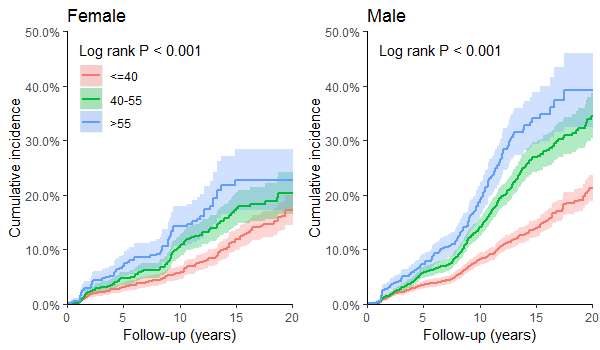


| **Year** | **0** | **5** | **10** | **15** | **20** |
| --- | --- | --- | --- | --- | --- |
| **Patients (n)** |  |  |  |  |  |
| **Female** |  |  |  |  |  |
| <=40 | 2,072 | 1,331 | 1,068 | 564 | 296 |
| 41-55 | 1,634 | 888 | 646 | 249 | 83 |
| >55 | 783 | 346 | 201 | 76 | 11 |
| **Male** |  |  |  |  |  |
| <=40 | 3,110 | 2,277 | 1,831 | 873 | 461 |
| 41-55 | 2,506 | 1,633 | 1,177 | 314 | 107 |
| >55 | 1,159 | 656 | 414 | 89 | 19 |
| **Cumulative incidence rate (%) (95% CI)** | | |  |  |  |
| **Female** |  |  |  |  |  |
| <=40 | 0.00% | 3.17% (2.32-4.01) | 5.72%  (4.52- 6.93) | 11.83%  (9.84-13.82) | 17.16%  (14.40-19.91) |
| 41-55 | 0.00% | 4.68%  (3.47- 5.89) | 10.52%  (8.54-12.50) | 17.22%  (14.27-20.17) | 20.34%  (16.50-24.18) |
| >55 | 0.00% | 6.87%  (4.70- 9.05) | 14.21%  (10.56-17.86) | 22.77%  (17.13-28.40) | 22.77%  (17.13-28.40) |
| **Male** |  |  |  |  |  |
| <=40 | 0.00% | 3.54%  (2.84-4.25) | 8.21%  (7.10-9.33) | 13.87%  (12.27-15.47) | 21.18%  (18.82-23.54) |
| 41-55 | 0.00% | 5.81%  (4.78-6.84) | 14.11%  (12.44-15.79) | 26.95%  (24.15-29.75) | 34.53%  (30.37-38.69) |
| >55 | 0.00% | 7.37%  (5.65-9.09) | 18.92%  (15.94-21.90) | 34.04%  (29.25-38.82) | 39.27%  (32.46-46.07) |

**Supplementary Figure 3. Cumulative incidence rates of spontaneous HBsAg seroclearance by HBeAg status, stratified by: (A) age, (B) sex, (C) HBV DNA, (D) qHBsAg, and (E) genotype**

**(A) Age**


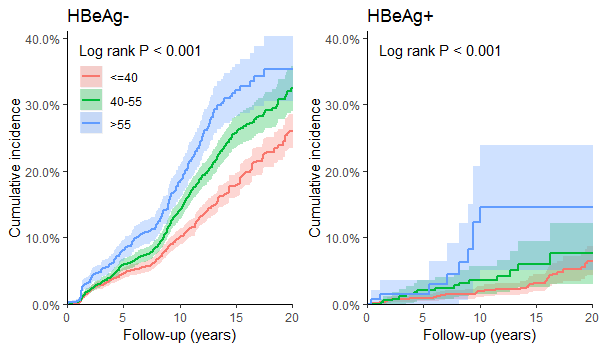


| **Year** | **0** | **5** | **10** | | **15** | | **20** |
| --- | --- | --- | --- | --- | --- | --- | --- |
| **Patients (n)** | | |  | |  | |  |
| **HBeAg (+)** |  |  |  | |  | |  |
| <=40 | 1,341 | 1,023 | 837 | | 476 | | 294 |
| 41-55 | 426 | 286 | 215 | | 80 | | 32 |
| >55 | 141 | 73 | 38 | | 15 | | 3 |
| **HBeAg (-)** |  |  |  | |  | |  |
| <=40 | 3,468 | 2,292 | 1,826 | | 812 | | 414 |
| 41-55 | 3,546 | 2,141 | 1,554 | | 460 | | 150 |
| >55 | 1,696 | 884 | 564 | | 148 | | 27 |
| **Cumulative incidence rate (%) (95% CI)** | | | |  | |  |  |
| **HBeAg (+)** |  |  |  | |  | |  |
| <=40 | 0.00% | 0.92%  (0.38-1.47) | 1.88% (1.06-2.71) | | 3.20%  (1.97-4.43) | | 6.51% (4.37-8.66) |
| 41-55 | 0.00% | 2.09%  (0.55-3.63) | 3.62%  (1.49-5.75) | | 6.03%  (2.57-9.49) | | 7.60% (3.02-12.18) |
| >55 | 0.00% | 1.54%  (0.00-3.66) | 14.53%  (5.14-23.91) | | 14.53%  (5.14-23.91) | | 14.53% (5.14-23.91) |
| **HBeAg (-)** |  |  |  | |  | |  |
| <=40 | 0.00% | 4.66%  (3.88-5.44) | 10.02% (8.82-11.21) | | 17.86%  (16.08-19.64) | | 25.97% (23.43-28.52) |
| 41-55 | 0.00% | 5.95% (5.06-6.84) | 14.18% (12.72-15.63) | | 25.74%  (23.43-28.06) | | 32.44% (29.05-35.83) |
| >55 | 0.00% | 8.05%  (6.53-9.56) | 18.30%  (15.81-20.79) | | 32.20%  (28.26-36.13) | | 35.35% (30.45-40.25) |

**(B) Sex**


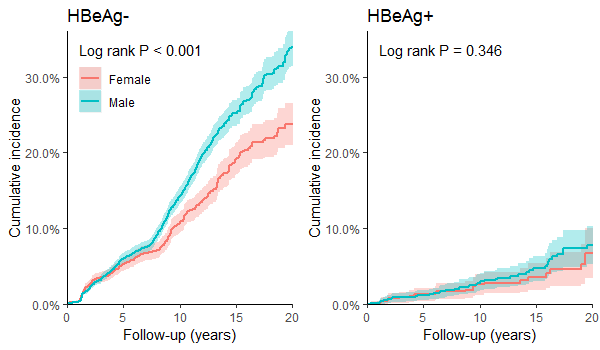


| **Year** | **0** | **5** | **10** | | **15** | | **20** | |
| --- | --- | --- | --- | --- | --- | --- | --- | --- |
| **Patients (n)** |  |  |  | |  | |  | |
| **HBeAg (+)** |  |  |  | |  | |  | |
| Female | 764 | 519 | 411 | | 222 | | 116 | |
| Male | 1,144 | 863 | 679 | | 349 | | 213 | |
| **HBeAg (-)** |  |  |  | |  | |  | |
| Female | 3,434 | 1,838 | 1,349 | | 578 | | 242 | |
| Male | 5,276 | 3,479 | 2,595 | | 842 | | 349 | |
| **Cumulative incidence rate (%) (95% CI)** | | | |  | |  | |  |
| **HBeAg (+)** |  |  |  | |  | |  | |
| Female | 0.00% | 1.26%  (0.39-2.14) | 2.57%  (1.22-3.92) | | 3.59%  (1.81-5.36) | | 6.78%  (3.48-10.08) | |
| Male | 0.00% | 1.19%  (0.52-1.87) | 2.97%  (1.83-4.11) | | 4.71%  (3.06-6.37) | | 7.75%  (5.22-10.29) | |
| **HBeAg (-)** |  |  |  | |  | |  | |
| Female | 0.00% | 5.27%  (4.39-6.14) | 10.70%  (9.33-12.06) | | 19.17%  (17.05-21.28) | | 23.75%  (21.01-26.50) | |
| Male | 0.00% | 6.07%  (5.35-6.78) | 14.30%  (13.16-15.44) | | 25.19%  (23.47-26.91) | | 33.91%  (31.37-36.44) | |

**(C) HBV DNA (IU/mL)**

**
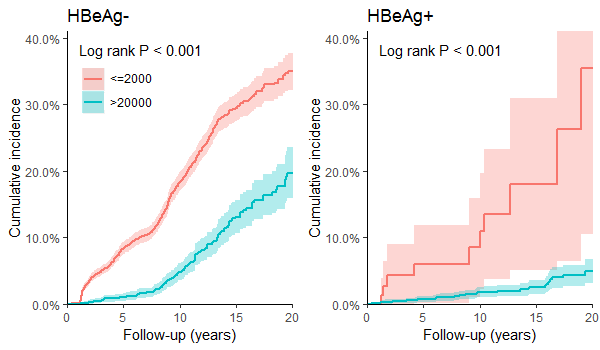
**

| **Year** | **0** | **5** | **10** | **15** | **20** |
| --- | --- | --- | --- | --- | --- |
| **Patients (n)** |  |  |  |  |  |
| **HBeAg (+)** |  |  |  |  |  |
| ≤2,000 | 84 | 50 | 36 | 16 | 6 |
| >20,000 | 1,445 | 1,119 | 925 | 471 | 270 |
| **HBeAg (-)** |  |  |  |  |  |
| ≤2,000 | 4,266 | 2,581 | 1,912 | 557 | 192 |
| >20,000 | 1,574 | 1,124 | 889 | 369 | 155 |
| **Cumulative incidence rate (%) (95% CI)** | | |  |  |  |
| **HBeAg (+)** |  |  |  |  |  |
| ≤2,000 | 0.00% | 6.03%  (0.21-11.84) | 10.97% (2.25-19.69) | 18.07% (5.17-30.96) | 35.48% (10.44-60.52) |
| >20,000 | 0.00% | 0.70% (0.24-1.16) | 1.76% (0.99-2.53) | 2.56% (1.51-3.61) | 4.94% (3.08-6.80) |
| **HBeAg (-)** |  |  |  |  |  |
| ≤2,000 | 0.00% | 8.38% (7.44-9.31) | 18.33% (16.90-19.76) | 29.47% (27.43-31.52) | 34.97% (32.19-37.74) |
| >20,000 | 0.00% | 1.09% (0.52-1.66) | 4.82% (3.53-6.11) | 13.12% (10.62-15.63) | 19.74% (15.90-23.57) |

**(D)** **qHBsAg (IU/mL)
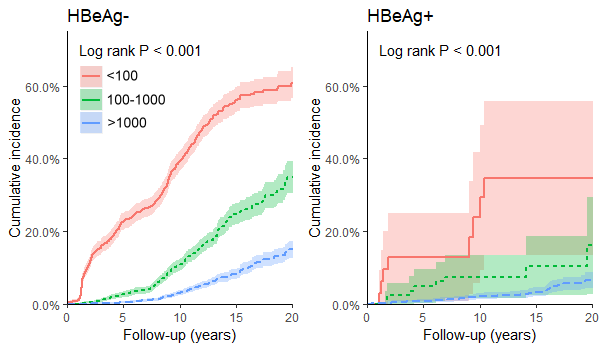
**

| **Year** | **0** | **5** | **10** | **15** | **20** |
| --- | --- | --- | --- | --- | --- |
| **Patients (n)** |  |  |  |  |  |
| **HBeAg (+)** |  |  |  |  |  |
| **qHBsAg (IU/mL)** |  |  |  |  |  |
| < 100 | 33 | 21 | 13 | 5 | 2 |
| 100-1000 | 100 | 76 | 58 | 27 | 12 |
| > 1000 | 1,333 | 1,088 | 913 | 487 | 287 |
| **HBeAg (-)** |  |  |  |  |  |
| **qHBsAg (IU/mL)** |  |  |  |  |  |
| < 100 | 1,412 | 867 | 614 | 147 | 46 |
| 100-1000 | 1,805 | 1,378 | 1,123 | 367 | 109 |
| > 1000 | 2,859 | 2,260 | 1,963 | 803 | 396 |
| **Cumulative incidence rate (%) (95% CI)** | | |  |  |  |
| **HBeAg (+)** |  |  |  |  |  |
| **qHBsAg (IU/mL)** |  |  |  |  |  |
| < 100 | 0.00% | 12.95% (0.88-25.01) | 29.27% (9.44-49.10) | 34.71% (13.52-55.90) | 34.71% (13.52-55.90) |
| 100-1000 | 0.00% | 4.88% (0.18-9.58) | 7.53% (1.69-13.37) | 10.51% (2.38-18.64) | 16.10% (2.75-29.46) |
| > 1000 | 0.00% | 0.82% (0.31-1.33) | 2.20% (1.32-3.07) | 3.33% (2.12-4.55) | 6.57% (4.46-8.67) |
| **HBeAg (-)** |  |  |  |  |  |
| **qHBsAg (IU/mL)** |  |  |  |  |  |
| < 100 | 0.00% | 22.70% (20.37-25.03) | 39.35%  (36.51-42.18) | 56.16%  (52.69-59.62) | 60.76%  (56.50-65.03) |
| 100-1000 | 0.00% | 2.68%  (1.87-3.49) | 10.76%  (9.12-12.39) | 24.82%  (21.93-27.71) | 34.86%  (30.37-39.35) |
| > 1000 | 0.00% | 0.62%  (0.31-0.93) | 3.13%  (2.40-3.86) | 8.24%  (6.87-9.62) | 14.97%  (12.63-17.31) |

**(E) Genotype**

**
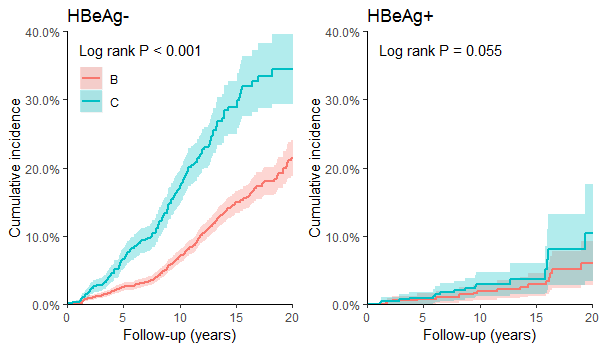
**

| **Year** | **0** | **5** | **10** | **15** | **20** |
| --- | --- | --- | --- | --- | --- |
| **Patients (n)** |  |  |  |  |  |
| **HBeAg (+)** |  |  |  |  |  |
| **Genotype** |  |  |  |  |  |
| B | 598 | 486 | 429 | 221 | 91 |
| C | 481 | 375 | 285 | 82 | 32 |
| **HBeAg (-)** |  |  |  |  |  |
| **Genotype** |  |  |  |  |  |
| B | 3,301 | 2,594 | 2,219 | 814 | 249 |
| C | 1,335 | 830 | 601 | 141 | 46 |
| **Cumulative incidence rate (%) (95% CI)** | | |  |  |  |
| **HBeAg (+)** |  |  |  |  |  |
| **Genotype** |  |  |  |  |  |
| B | 0.00% | 0.78% (0.02-1.54) | 1.86% (0.65-3.07) | 2.89% (1.21-4.57) | 5.99% (2.79-9.18) |
| C | 0.00% | 0.92% (0.02-1.81) | 2.99% (1.23-4.74) | 3.73% (1.46-6.01) | 10.47% (3.43-17.51) |
| **HBeAg (-)** |  |  |  |  |  |
| **Genotype** |  |  |  |  |  |
| B | 0.00% | 2.53% (1.96-3.10) | 6.98% (6.01-7.95) | 14.90% (13.27-16.54) | 21.43% (18.76-24.10) |
| C | 0.00% | 6.65% (5.14-8.15) | 16.97% (14.47-19.48) | 28.81% (24.99-32.63) | 34.51% (29.37-39.65) |

**Supplementary Figure 4. Cumulative incidence rates of spontaneous HBsAg seroclearance rate by genotype, stratified by: (A) age, (B) sex, (C) qHBsAg, and (D) infection phase**

**(A) Age**

**
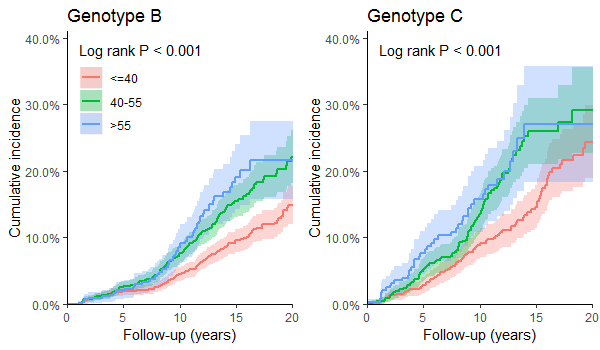
**

| **Year** | **0** | **5** | **10** | **15** | **20** |
| --- | --- | --- | --- | --- | --- |
| **Patients (n)** |  |  |  |  |  |
| **Genotype B** |  |  |  |  |  |
| <=40 | 1,785 | 1,507 | 1,330 | 587 | 220 |
| 41-55 | 1,545 | 1,191 | 1,025 | 370 | 111 |
| >55 | 604 | 414 | 318 | 96 | 16 |
| **Genotype C** |  |  |  |  |  |
| <=40 | 864 | 674 | 533 | 194 | 59 |
| 41-55 | 799 | 534 | 387 | 81 | 30 |
| >55 | 362 | 191 | 120 | 28 | 5 |
| **Cumulative incidence rate (%) (95% CI)** | | |  |  |  |
| **Genotype B** |  |  |  |  |  |
| <=40 | 0.00% | 1.96% (1.29-2.64) | 4.48% (3.45-5.52) | 9.56% (7.84-11.28) | 14.86% (12.06-17.66) |
| 41-55 | 0.00% | 2.65% (1.78-3.52) | 7.60% (6.11-9.09) | 15.46% (13.01-17.91) | 22.15% (18.17-26.12) |
| >55 | 0.00% | 2.07% (0.79-3.34) | 8.57% (5.83-11.32) | 19.14% (14.22-24.07) | 21.61% (15.72-27.49) |
| **Genotype C** |  |  |  |  |  |
| <=40 | 0.00% | 2.84% (1.67-4.01) | 8.87% (6.73-11.01) | 14.63% (11.41-17.84) | 24.40% (18.96-29.83) |
| 41-55 | 0.00% | 5.19% (3.48-6.90) | 13.46% (10.57-16.35) | 25.93% (20.97-30.90) | 29.15% (22.64-35.66) |
| >55 | 0.00% | 7.70% (4.49-10.91) | 15.77% (10.75-20.78) | 27.08% (18.33-35.82) | 27.08% (18.33-35.82) |

**(B) Sex**

**
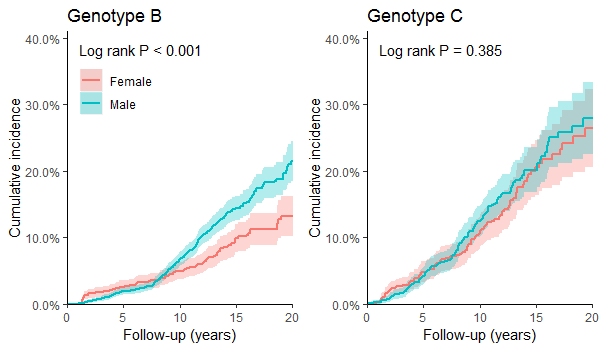
**

| **Year** | **0** | **5** | **10** | **15** | **20** |
| --- | --- | --- | --- | --- | --- |
| **Patients (n)** |  |  |  |  |  |
| **Genotype B** |  |  |  |  |  |
| **Sex** |  |  |  |  |  |
| Female | 1,460 | 975 | 863 | 419 | 134 |
| Male | 2,474 | 2,137 | 1,810 | 634 | 213 |
| **Genotype C** |  |  |  |  |  |
| **Sex** |  |  |  |  |  |
| Female | 873 | 608 | 470 | 141 | 46 |
| Male | 1,152 | 791 | 570 | 162 | 48 |
| **Cumulative incidence rate (%) (95% CI)** | | |  |  |  |
| **Genotype B** |  |  |  |  |  |
| **Sex** |  |  |  |  |  |
| Female | 0.00% | 2.62% (1.71-3.54) | 5.01% (3.69-6.32) | 9.93% (7.82-12.04) | 13.24% (10.22-16.27) |
| Male | 0.00% | 2.02% (1.44-2.59) | 6.71% (5.65-7.78) | 14.40% (12.60-16.21) | 21.44% (18.44-24.43) |
| **Genotype C** |  |  |  |  |  |
| **Sex** |  |  |  |  |  |
| Female | 0.00% | 4.85% (3.29-6.40) | 10.71% (8.30-13.12) | 20.55% (16.44-24.67) | 26.51% (20.64-32.37) |
| Male | 0.00% | 4.28% (2.99-5.56) | 12.44% (10.13-14.74) | 20.19% (16.79-23.59) | 27.92% (22.55-33.29) |

**(C) qHBsAg**

**
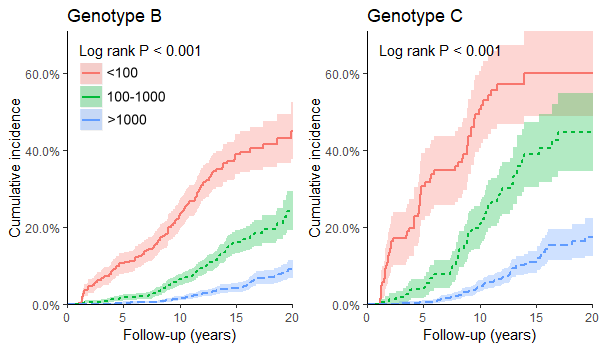
**

| **Year** | **0** | **5** | **10** | **15** | **20** |
| --- | --- | --- | --- | --- | --- |
| **Patients (n)** |  |  |  |  |  |
| **Genotype B** |  |  |  |  |  |
| **qHBsAg** |  |  |  |  |  |
| < 100 | 567 | 413 | 321 | 95 | 26 |
| 100-1,000 | 1,170 | 966 | 835 | 296 | 76 |
| > 1,000 | 1,835 | 1,618 | 1,472 | 643 | 238 |
| **Genotype C** |  |  |  |  |  |
| **qHBsAg** |  |  |  |  |  |
| < 100 | 126 | 69 | 41 | 10 | 2 |
| 100-1,000 | 246 | 205 | 145 | 38 | 14 |
| > 1,000 | 971 | 832 | 689 | 174 | 62 |
| **Cumulative incidence rate (%) (95% CI)** | | |  |  |  |
| **Genotype B** |  |  |  |  |  |
| **qHBsAg** |  |  |  |  |  |
| < 100 | 0.00% | 10.64% (7.92-13.35) | 23.18%  (19.34-27.03) | 38.96%  (33.69-44.22) | 45.11%  (37.76-52.46) |
| 100-1,000 | 0.00% | 1.72%  (0.93-2.51) | 6.37%  (4.83-7.91) | 16.17%  (13.21-19.13) | 24.27%  (19.19-29.35) |
| > 1,000 | 0.00% | 0.36%  (0.07-0.66) | 1.51%  (0.91-2.12) | 4.25%  (3.10-5.40) | 9.02%  (6.68-11.37) |
| **Genotype C** |  |  |  |  |  |
| **qHBsAg** |  |  |  |  |  |
| < 100 | 0.00% | 30.66%  (21.95-39.37) | 50.66%  (40.60-60.72) | 60.06%  (48.95-71.16) | 60.06%  (48.95-71.16) |
| 100-1,000 | 0.00% | 4.02%  (1.44-6.60) | 19.89%  (14.40-25.37) | 38.91%  (30.40-47.41) | 44.77%  (34.71-54.84) |
| > 1,000 | 0.00% | 0.57%  (0.07-1.06) | 4.64%  (3.18-6.11) | 10.86%  (8.06-13.66) | 17.55%  (12.60-22.50) |

**(D) Infection phase.**

**
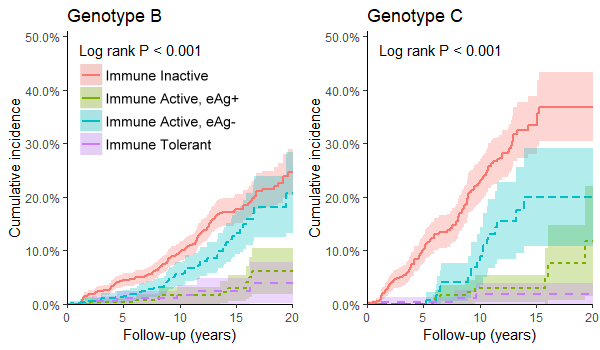
**

| **Year** | **0** | **5** | **10** | **15** | **20** |
| --- | --- | --- | --- | --- | --- |
| **Patients (n)** |  |  |  |  |  |
| **Genotype B** |  |  |  |  |  |
| **Infection phase** |  |  |  |  |  |
| Immune inactive | 1,262 | 978 | 839 | 301 | 91 |
| Immune active, eAg+ | 283 | 245 | 224 | 120 | 47 |
| Immune active, eAg- | 495 | 414 | 351 | 136 | 27 |
| Immune tolerant | 243 | 187 | 160 | 75 | 29 |
| **Genotype C** |  |  |  |  |  |
| **Infection phase** |  |  |  |  |  |
| Immune inactive | 611 | 393 | 289 | 61 | 26 |
| Immune active, eAg+ | 220 | 186 | 142 | 51 | 20 |
| Immune active, eAg- | 202 | 130 | 92 | 27 | 4 |
| Immune tolerant | 225 | 170 | 131 | 23 | 8 |
| **Cumulative incidence rate (%) (95% CI)** | | |  |  |  |
| **Genotype B** |  |  |  |  |  |
| **Infection phase** |  |  |  |  |  |
| Immune inactive | 0.00% | 4.50% (3.28-5.72) | 9.78%  (7.97-11.59) | 17.80%  (15.11-20.50) | 24.65%  (20.37-28.93) |
| Immune active, eAg+ | 0.00% | 0.39%  (0.00-1.14) | 1.63%  (0.04-3.21) | 3.00%  (0.54-5.46) | 6.19%  (1.90-10.48) |
| Immune active, eAg- | 0.00% | 1.55%  (0.41-2.69) | 5.56%  (3.34-7.78) | 13.30%  (9.26-17.34) | 20.78%  (13.23-28.34) |
| Immune tolerant | 0.00% | 1.03%  (0.00-2.45) | 1.64%  (0.00-3.48) | 2.41%  (0.03-4.79) | 3.98%  (0.11-7.86) |
| **Genotype C** |  |  |  |  |  |
| **Infection phase** |  |  |  |  |  |
| Immune inactive | 0.00% | 10.23%  (7.60-12.86) | 22.83%  (18.90-26.76) | 33.48%  (28.01-38.94) | 36.86%  (30.45-43.27) |
| Immune active, eAg+ | 0.00% | 0.00% | 2.94%  (0.39-5.49) | 2.94%  (0.39-5.49) | 11.75%  (1.42-22.09) |
| Immune active, eAg- | 0.00% | 0.00% | 7.96%  (2.92-13.00) | 19.97%  (10.88-29.06) | 19.97%  (10.88-29.06) |
| Immune tolerant | 0.00% | 0.46%  (0.00-1.36) | 1.81%  (0.00-3.87) | 1.81%  (0.00-3.87) | 1.81%  (0.00-3.87) |

**Supplementary Figure 5. Cumulative incidence rates of spontaneous HBsAg seroclearance by infection phase, stratified by: (A) age, (B) sex, and (C) qHBsAg**

**(A) Age**

**
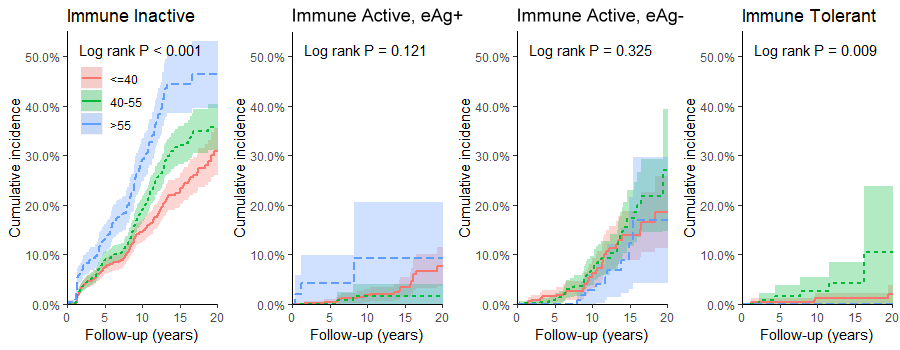
**

| **Year** | **0** | **5** | **10** | **15** | **20** |
| --- | --- | --- | --- | --- | --- |
| **Patients (n)** |  |  |  |  |  |
| **Immune inactive** |  |  |  |  |  |
| <=40 | 1,288 | 878 | 717 | 238 | 87 |
| 41-55 | 1,332 | 828 | 630 | 149 | 55 |
| >55 | 671 | 365 | 239 | 57 | 10 |
| **Immune active, eAg+** | |  |  |  |  |
| <=40 | 495 | 416 | 349 | 184 | 92 |
| 41-55 | 168 | 129 | 99 | 42 | 16 |
| >55 | 52 | 25 | 15 | 8 | 2 |
| **Immune active, eAg-** | |  |  |  |  |
| <=40 | 384 | 266 | 199 | 86 | 32 |
| 41-55 | 464 | 331 | 232 | 85 | 14 |
| >55 | 224 | 127 | 88 | 25 | 2 |
| **Immune tolerant** |  |  |  |  |  |
| <=40 | 434 | 358 | 303 | 164 | 112 |
| 41-55 | 160 | 114 | 90 | 22 | 8 |
| >55 | 51 | 28 | 20 | 6 | 1 |
| **Cumulative incidence rate (%) (95% CI)** | | |  |  |  |
| **Immune inactive** |  |  |  |  |  |
| <=40 | 0.00% | 7.51% (5.95-9.07) | 14.65%  (12.43-16.87) | 22.62%  (19.55-25.69) | 30.80%  (26.07-35.53) |
| 41-55 | 0.00% | 8.80%  (7.10-10.50) | 19.20%  (16.65-21.75) | 32.00%  (28.20-35.80) | 35.67%  (31.04-40.29) |
| >55 | 0.00% | 13.17%  (10.29-16.05) | 28.83%  (24.49-33.17) | 44.41%  (38.46-50.36) | 46.26%  (39.46-53.07) |
| **Immune active, eAg+** | |  |  |  |  |
| <=40 | 0.00% | 0.45%  (0.00-1.08) | 1.72%  (0.45-2.99) | 3.39%  (1.32-5.45) | 7.63%  (3.71-11.55) |
| 41-55 | 0.00% | 0.00% | 1.66%  (0.00-3.96) | 1.66%  (0.00-3.96) | 1.66%  (0.00-3.96) |
| >55 | 0.00% | 4.19%  (0.00-9.95) | 9.23%  (0.00-20.52) | 9.23%  (0.00-20.52) | 9.23%  (0.00-20.52) |
| **Immune active, eAg-** | |  |  |  |  |
| <=40 | 0.00% | 1.53%  (0.19-2.86) | 5.82%  (2.91-8.74) | 13.80%  (8.77-18.83) | 18.56%  (11.36-25.75) |
| 41-55 | 0.00% | 1.07%  (0.02-2.12) | 6.70%  (3.83-9.57) | 17.28%  (11.68-22.88) | 27.04%  (14.73-39.34) |
| >55 | 0.00% | 0.00% | 2.84%  (0.00-6.02) | 12.29%  (2.97-21.61) | 16.91%  (4.26-29.55) |
| **Immune tolerant** |  |  |  |  |  |
| <=40 | 0.00% | 0.47%  (0.00-1.12) | 1.11%  (0.01-2.21) | 1.11%  (0.01-2.21) | 1.94%  (0.00-3.89) |
| 41-55 | 0.00% | 1.59%  (0.00-3.79) | 2.52%  (0.00-5.36) | 4.17%  (0.00-8.44) | 10.56%  (0.00-23.70) |
| >55 | 0.00% | 0.00% | 0.00% | 0.00% | 0.00% |

**(B) Sex**

**
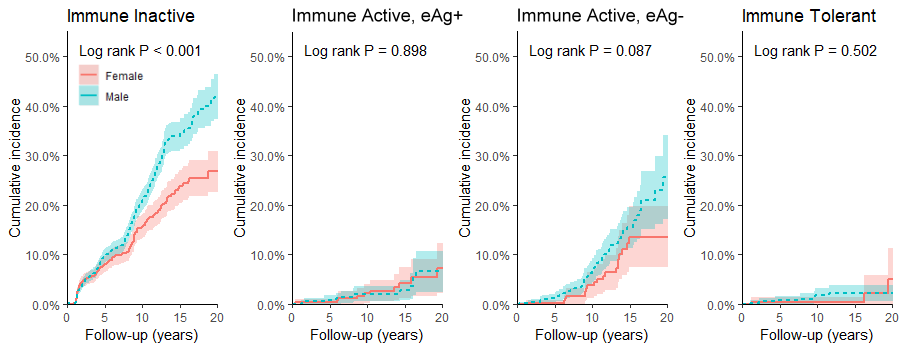
**

| **Year** | **0** | **5** | **10** | **15** | **20** |
| --- | --- | --- | --- | --- | --- |
| **Patients (n)** |  |  |  |  |  |
| **Immune inactive** |  |  |  |  |  |
| Female | 1,384 | 765 | 590 | 221 | 78 |
| Male | 1,907 | 1,306 | 996 | 223 | 74 |
| **Immune active, eAg+** | |  |  |  |  |
| Female | 300 | 239 | 197 | 100 | 47 |
| Male | 415 | 331 | 266 | 134 | 63 |
| **Immune active, eAg-** |  |  |  |  |  |
| Female | 383 | 236 | 165 | 66 | 21 |
| Male | 689 | 488 | 354 | 130 | 27 |
| **Immune tolerant** |  |  |  |  |  |
| Female | 228 | 154 | 131 | 65 | 32 |
| Male | 417 | 346 | 282 | 127 | 89 |
| **Cumulative incidence rate (%) (95% CI)** | | |  |  |  |
| **Immune inactive** |  |  |  |  |  |
| Female | 0.00% | 8.07% (6.42-9.71) | 15.70%  (13.28-18.11) | 23.69%  (20.33-27.04) | 26.73%  (22.66-30.80) |
| Male | 0.00% | 9.72%  (8.29-11.15) | 20.96%  (18.87-23.05) | 33.77%  (30.81-36.73) | 41.81%  (37.34-46.29) |
| **Immune active, eAg+** | |  |  |  |  |
| Female | 0.00% | 0.33%  (0.00-0.99) | 2.15%  (0.27-4.03) | 4.30%  (1.22-7.38) | 7.23%  (2.11-12.35) |
| Male | 0.00% | 0.81%  (0.00-1.73) | 2.06%  (0.54-3.58) | 2.73%  (0.73-4.73) | 6.55%  (2.39-10.70) |
| **Immune active, eAg-** |  |  |  |  |  |
| Female | 0.00% | 0.29%  (0.00-0.86) | 3.79%  (1.17-6.41) | 13.52%  (7.37-19.67) | 13.52%  (7.37-19.67) |
| Male | 0.00% | 1.41%  (0.44-2.39) | 6.58%  (4.27-8.88) | 15.46%  (11.35-19.57) | 25.61%  (17.21-34.01) |
| **Immune tolerant** |  |  |  |  |  |
| Female | 0.00% | 0.46%  (0.00-1.36) | 0.46%  (0.00-1.36) | 0.46%  (0.00-1.36) | 4.95%  (0.00-11.32) |
| Male | 0.00% | 0.79%  (0.00-1.68) | 1.77%  (0.35-3.20) | 2.24%  (0.56-3.92) | 2.24%  (0.56-3.92) |

**(C) qHBsAg**

**
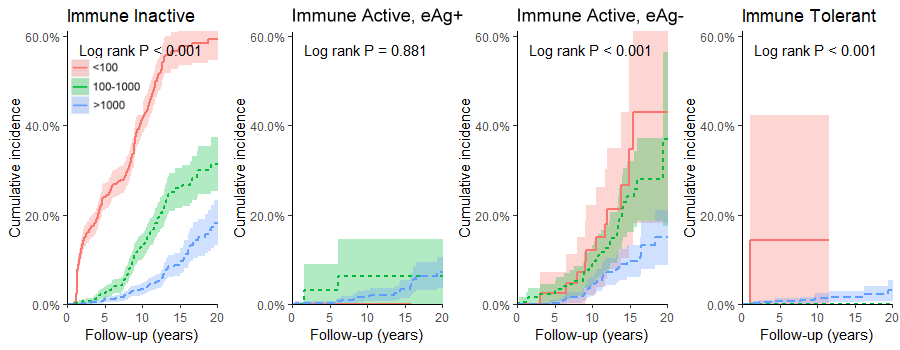
**

| **Year** | **0** | **5** | **10** | **15** | **20** |
| --- | --- | --- | --- | --- | --- |
| **Patients (n)** |  |  |  |  |  |
| **Immune inactive** |  |  |  |  |  |
| < 100 | 946 | 564 | 396 | 80 | 28 |
| 100-1000 | 761 | 562 | 466 | 135 | 39 |
| > 1000 | 1,013 | 761 | 681 | 212 | 85 |
| **Immune active, eAg+** | |  |  |  |  |
| < 100 | 8 | 7 | 4 | 1 | 0 |
| 100-1000 | 38 | 32 | 26 | 14 | 6 |
| > 1000 | 484 | 439 | 388 | 205 | 104 |
| **Immune active, eAg-** | |  |  |  |  |
| < 100 | 44 | 41 | 35 | 8 | 1 |
| 100-1000 | 184 | 169 | 141 | 53 | 5 |
| > 1000 | 365 | 339 | 294 | 120 | 40 |
| **Immune tolerant** |  |  |  |  |  |
| < 100 | 7 | 2 | 2 | 0 | 0 |
| 100-1000 | 27 | 19 | 14 | 3 | 1 |
| > 1000 | 544 | 451 | 384 | 183 | 116 |
| **Cumulative incidence rate (%) (95% CI)** | | |  |  |  |
| **Immune inactive** |  |  |  |  |  |
| < 100 | 0.00% | 24.02% (21.10-26.93) | 41.50%  (37.99-45.01) | 57.59%  (53.34-61.85) | 59.31%  (54.58-64.03) |
| 100-1000 | 0.00% | 2.56%  (1.31-3.80) | 13.03%  (10.27-15.79) | 25.95%  (21.54-30.35) | 31.31%  (25.35-37.27) |
| > 1000 | 0.00% | 1.12%  (0.39-1.85) | 3.40%  (2.11-4.69) | 8.82%  (6.28-11.36) | 18.13%  (13.10-23.17) |
| **Immune active, eAg+** | |  |  |  |  |
| < 100 | 0.00% | 0.00% | 0.00% | 0.00% | NA |
| 100-1000 | 0.00% | 3.03%  (0.00-8.97) | 6.16%  (0.00-14.56) | 6.16%  (0.00-14.56) | 6.16%  (0.00-14.56) |
| > 1000 | 0.00% | 0.21%  (0.00-0.62) | 1.85%  (0.58-3.13) | 3.36%  (1.41-5.31) | 7.08%  (3.56-10.60) |
| **Immune active, eAg-** | |  |  |  |  |
| < 100 | 0.00% | 2.38%  (0.00-7.05) | 12.03%  (2.01-22.05) | 34.73%  (13.14-56.32) | 42.89%  (18.14-67.64) |
| 100-1000 | 0.00% | 2.79%  (0.37-5.21) | 8.24%  (4.08-12.39) | 24.06%  (16.09-32.02) | 36.86%  (17.44-56.28) |
| > 1000 | 0.00% | 0.29%  (0.00-0.86) | 3.98%  (1.86-6.11) | 9.58%  (5.92-13.25) | 14.90%  (8.69-21.11) |
| **Immune tolerant** |  |  |  |  |  |
| < 100 | 0.00% | 14.29%  (0.00-42.29) | 14.29%  (0.00-42.29) | NA | NA |
| 100-1000 | 0.00% | 0.00% | 0.00% | 0.00% | 0.00% |
| > 1000 | 0.00% | 0.61%  (0.00-1.31) | 1.34%  (0.27-2.42) | 1.67%  (0.42-2.91) | 3.05%  (0.77-5.34) |
